# Oral Health in Early and Advanced Stages (1-4) of Chronic Kidney Disease: A Systematic Review and Meta-Analysis

**DOI:** 10.1016/j.identj.2026.109610

**Published:** 2026-05-18

**Authors:** Christian Niederau, Asia Azad Ali, Saida Chahboun, Sahir Kalim, Mariano Rodriguez Portillo, Rafael Kramann, Ulrike Schulze-Späte, Stefan Wolfart, Joachim Jankowski, Michael Wolf, Rogerio B. Craveiro

**Affiliations:** a Department of Orthodontics, Medical Faculty, RWTH-Aachen University, Aachen, Germany; b Department of Medicine, Division of Nephrology, Massachusetts General Hospital and Harvard Medical School, Boston, Massachusetts, USA; c Maimonides Institute for Biomedical Research (IMIBIC), Cordoba, Spain; d Department of Medicine, School of Medicine, University of Cordoba, Cordoba, Spain; e Nephrology Service, Reina Sofia University Hospital, Cordoba, Spain; f Division of Nephrology and Clinical Immunology, Medical Faculty, RWTH-Aachen University, Aachen, Germany; g Section of Geriodontics, Department of Conservative Dentistry and Periodontics, University Hospital Jena, Jena, Germany; h Department of Prosthodontics and Biomaterials, RWTH Aachen University, Germany; i Institute of Molecular Cardiovascular Research, Medical Faculty, RWTH-Aachen University, Aachen, Germany; j Aachen-Maastricht Institute for Cardiorenal Disease (AMICARE), University Hospital RWTH Aachen, Aachen, Germany; k Experimental Vascular Pathology, Cardiovascular Research Institute Maastricht (CARIM), University of Maastricht, Maastricht, The Netherlands

**Keywords:** Dental interventions, Chronic kidney disease, Early and advanced stages, Oral manifestation, Oral health, BOP bleeding of probing, Inflammation

## Abstract

It is essential to identify oral health changes in the early stages of chronic kidney disease (CKD) to prevent oral degeneration. The effects of early-stage CKD on the periodontium and the manifestation of associated oral symptoms remain poorly understood. Periodontitis is well documented in late-stage CKD. These changes represent a considerable burden for patients, leading to consequences like tooth loss and stomatognathic disorders. This study evaluates oral health parameters in patients with early- and advanced-stage CKD (stages 1-4), to identify potential changes in oral health and provide important insights into CKD-specific dental treatment. We performed a comprehensive literature search of the databases ‘Pubmed’, ‘Web of Science’, ‘ClinicalTrials.gov’, and ‘Cochrane’ up to June 2025. Of 1,584 search results, 24 studies met the inclusion criteria. The meta-analysis revealed that patients with early- and advanced-stage CKD exhibited significantly higher levels of plaque and calculus accumulation compared to healthy patients. These patients experienced pronounced gingival inflammation, elevated gingival index scores, and increased ‘bleeding on probing’ levels. They demonstrated significantly higher salivary pH levels and reduced salivary flow rates. There is a strong relationship between early and advanced-stage CKD and increased oral inflammation, plaque accumulation, and reduced saliva flow rate. To prevent the dental health of these patients from deteriorating, it is crucial to refer them to a dentist rapidly during the progression of the disease. Based on their understanding of the impact of CKD on oral health, the dentist will then determine a patient-specific recall interval.

## Introduction

Chronic kidney disease (CKD) is a major global health challenge linked with a variety of systemic complications^1^ CKD affects over 10% of the world's population, equivalent to more than 800 million people, and is a leading cause of morbidity and mortality, which will even increase further in the future.^2^^,^^3^ The capacity of kidneys to extract harmful substances and excess fluids from the blood is progressively reduced, leading to an accumulation of uremic metabolites in the body.^4^

Numerous studies have demonstrated an association between CKD and oral health changes, such as periodontal disease, loss of attachment between tooth and alveolar bone and tooth loss.^5^, ^6^, ^7^, ^8^, ^9^, ^10^ Oral bone tissue changes, such as alveolar bone demineralisation, and oral soft tissue changes, such as gingival overgrowth and uraemic stomatitis, are also potential abnormalities associated with CKD and its drug treatment regimes.^11^ Severe renal insufficiency (stage 4-5) disrupts the balance of the stomatognathic system, increasing the incidence of common oral diseases, including periodontal disease and dental caries.^12^ In order to investigate the influence of early and advanced CKD on the development of irreversible damage to hard and soft oral tissue, which is already known to occur in patients with late-stage CKD, early detectable precursor stages of the changes were deliberately evaluated.

In general, oral health changes in CKD result from several underlying factors, including the saliva composition and flow rate and the inflammatory features of oral health.^13^^,^^14^ Altered oral parameters are verified by standardised procedures determining oral inflammation and including several measurements such as ‘bleeding on probing’, periodontal pocket depth, ginigival index, and attachment loss.^15^ An altered immune response in CKD likely impairs the body's ability to effectively reduce the amount of bacteria in the oral cavity,^16^^,^^17^ contributing to dysbiotic dental plaque accumulation and making it more challenging to manage and control onset of dental diseases.^18^^,^^19^ In addition, individuals with CKD often experience reduced saliva production due to changes in salivary composition,^20^^,^^21^ either as a result of CKD itself or as an effect of CKD medications.^22^ Since saliva is essential for neutralising oral acids and protecting the teeth, a deficiency in saliva increases the risk of caries.^23^ The rate of progression of dental disease is influenced by the severity of CKD, associated medications,^22^ and the patient's oral hygiene habits.^24^

In particular, oral problems associated with CKD are often only detected in late stages of CKD, when they have reached a serious stage^25^ and are therefore difficult to treat appropriately.

While many studies have examined the oral health changes of end-stage CKD (stage 5),^26^, ^27^, ^28^, ^29^, ^30^ the severity of oral health problems in the early and advanced stages of CKD (stage 1-4) has not been systematically analysed until now. Despite the importance of both early and advanced CKD for oral health deterioration and the need to provide necessary dental interventions, there is currently no specific overview or literature-based guidance for dental interventions at early and advanced CKD stages.

Therefore, this systematic review aims to provide a comprehensive overview of specific oral health changes in early and advanced stages of CKD compared to healthy individuals. The results are critically evaluated using dental index parameters to identify the oral health of patients with CKD at these stages to provide important insights into CKD-specific dental monitoring and treatment strategies, which are relevant for dentists and nephrology specialists.

## Material and methods

**Fig. 1:**
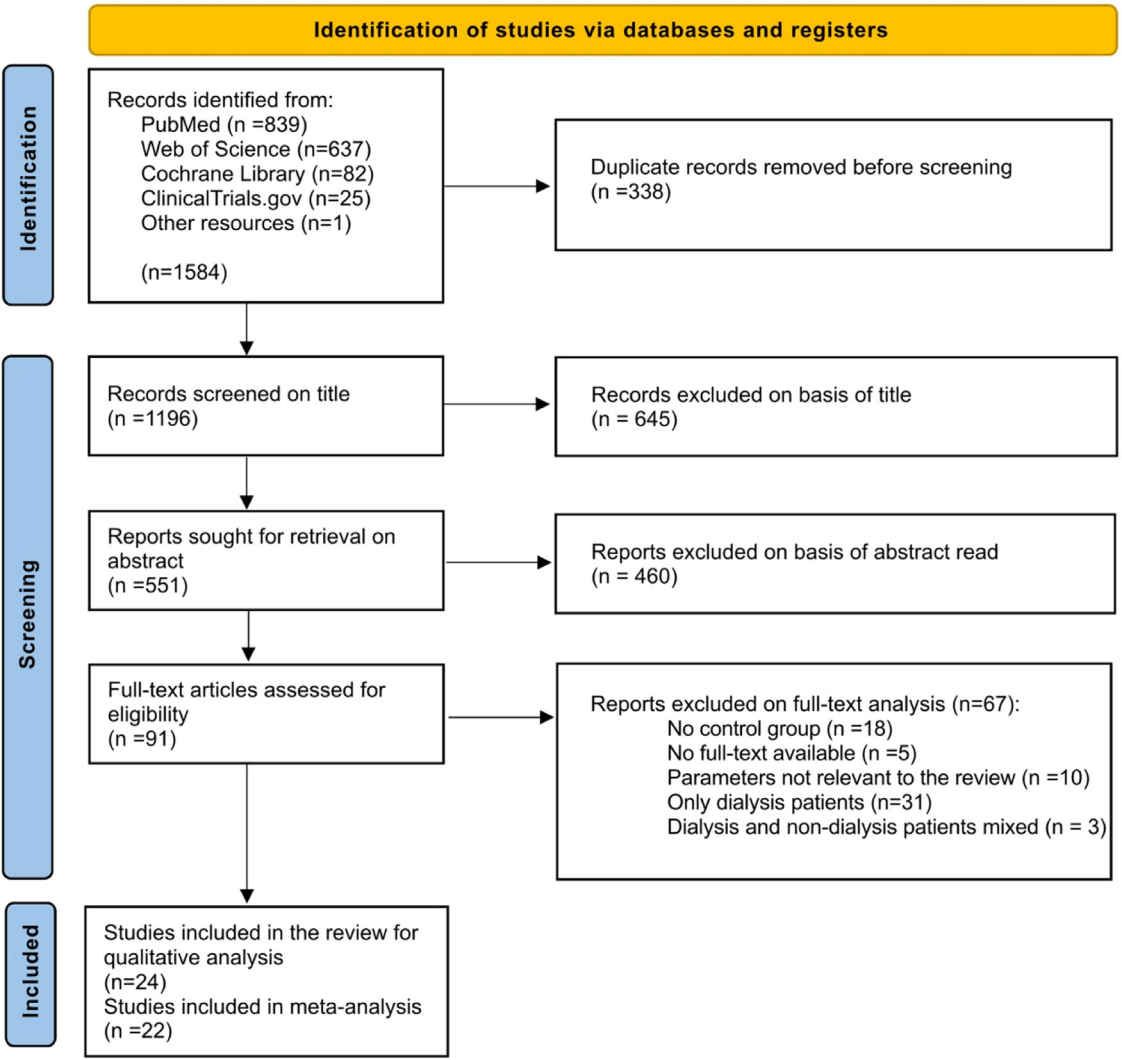
PRISMA flow chart describing the search strategy and study selection. Detailed information about reasons for exclusion is shown in Supplementary Table I.

This systematic review was recorded with the ‘International Prospective Register of Systematic Reviews’ (PROSPERO) under the protocol number CRD42024568036. The reporting followed the guidelines of the ‘Preferred Reporting Items for Systematic Reviews and Meta-Analyses’ (PRISMA) checklist.^31^ The PRISMA diagram is provided in Figure 1.

### Review question

The guiding question for the literature search was formulated using the PECO-S framework^32^^,^^33^: **Population (P)**: Patients with CKD in early stages and in advanced phase, stage 1-4, excluding dialysis patients or patients with 'end stage kidney disease, stage 5′; **Exposure (E)**: CKD; **Comparator (C):** patients without chronic kidney disease; **Outcome (O)**: Oral health (DMFT) index, plaque index (PI), oral hygiene index simplified (OHI-S), salivary pH, salivary flow rate, bleeding on probing (BOP), periodontal pocket depth (PPD) clinical attachment lost (CAL), gingival index (GI); **Study design (S):** clinical Studies with available full-text reports.

Detailed information about search strategies, selection of studies, data extraction and management, quality assessment of selected studies (Table 1) and statistical analysis is shown in the supplementary material.

**Table 1:** Systematic review search strategy.

Database	Search strategy
PubMed	("tooth decay" OR "delayed tooth eruption" OR "tooth eruption"[MeSH] OR "developmental defects of enamel"[MeSH] OR "congenital enamel hypoplasia" OR "dental enamel hypoplasia"[MeSH] OR "dental health surveys"[MeSH] OR "molar incisor hypomineralization" OR caries OR "dental caries"[MeSH] OR "bleeding on probing" OR BOP OR saliva OR "oral hygiene index"[MeSH] OR DMFT OR "dental plaque index"[MeSH] OR "periodontal index"[MeSH] OR "plaque index" OR gingivitis OR periodontitis OR "white spot" OR "loose tooth" OR "loose teeth" OR "tooth Loss" OR "tooth Loss"[MeSH] OR "teeth loss") AND ("chronic kidney disease" OR "chronic renal disease" OR "renal insufficiency, chronic" OR "renal insufficiency, chronic"[MeSH])
Web of Science	(ALL=("renal insufficiency chronic") OR (ALL="chronic kidney disease")) AND (ALL=("tooth decay" OR "delayed tooth eruption" OR "tooth eruption" OR "developmental defects of enamel" OR "congenital enamel hypoplasia" OR "dental enamel hypoplasia" OR "dental health surveys" OR "molar incisor hypomineralization" OR caries OR "dental caries" OR "bleeding on probing" OR BOP OR saliva OR "oral hygiene index" OR DMFT OR "dental plaque index" OR "periodontal index" OR "plaque index" OR gingivitis OR periodontitis OR "white spot" OR "loose tooth" OR "loose teeth" OR "tooth Loss" OR "tooth Loss" OR "teeth loss"))
Cochrane Library	"chronic kidney disease" AND ("tooth decay" OR "delayed tooth eruption" OR "tooth eruption" OR "developmental defects of enamel" OR "congenital enamel hypoplasia" OR "dental enamel hypoplasia" OR "dental health surveys" OR "molar incisor hypomineralization" OR caries OR "dental caries" OR "bleeding on probing" OR BOP OR saliva OR "oral hygiene index" OR DMFT OR "dental plaque index" OR "periodontal index" OR "plaque index" OR gingivitis OR periodontitis OR "white spot" OR "loose tooth" OR "loose teeth" OR "tooth Loss" OR "tooth Loss" OR "teeth loss")
ClinicalTrials.gov	"chronic kidney disease" and …
	- "tooth decay" OR "delayed tooth eruption" OR "tooth eruption"
	- "developmental defects of enamel" OR "congenital enamel hypoplasia" OR "dental enamel hypoplasia"
	- "dental health surveys" OR "molar incisor hypomineralization" OR caries OR "dental caries"
	- "bleeding on probing" OR BOP OR saliva OR "oral hygiene index" OR DMFT OR "dental plaque index" OR "periodontal index" OR "plaque index" OR gingivitis OR periodontitis OR "white spot"
	- "loose tooth" OR "loose teeth" OR "tooth Loss" OR "tooth Loss" OR "teeth loss"

### Search strategies

A systematic literature search was performed across 4 electronic databases, including PubMed of the US National Library, Web of Science, ClinicalTrials.gov, and Cochrane, without time restrictions up to January 2025.

An initial search strategy developed in PubMed was adapted for use in the other databases. Boolean operators (OR, AND) were applied in all databases to refine and narrow the searches, including relevant MeSH terms (Table 1). CKD stage was used as an exclusion criterion and not as a search criterion to avoid the risk of missing studies that evaluated the late CKD stage but also included early and advanced CKD stages in the analysis.

### Selection of studies

Title management was conducted electronically using EndNote 20 (Clarivate Analytics). Two investigators (AAA and SC) independently evaluated and examined the entire study process.

The review process occurred in 2 phases: first, titles and abstracts of all retrieved records were screened according to the following predefined inclusion criteria: English language, patients with CKD in early or advanced stage, retrospective and prospective studies, respectively, investigating oral health, females and males, patients: general population (all ethnicities, community-dwelling). If abstracts lacked clarity, full texts were reviewed. In the second step, all full-text articles selected initially were obtained and assessed based on the following exclusion criteria: patients undergoing dialysis or kidney transplantation, studies lacking a control group, insufficient clinical data on oral health parameters, or animal studies. Cohen’s kappa coefficient (κ) was calculated to measure the level of agreement between reviewers.

### Data extraction and management

Data extraction was carried out independently by 2 separate reviewers (A.A.A. and S.C.) to obtain relevant data regarding oral health-related parameters on CKD patients. As different countries use different methods to measure dental parameters, we have used the following parameters for international comparability: DMFT index for the assessing the number of decayed, filled, or missing teeth^34^; ‘plaque index’ (PI) by Silness and Loe^35^ for measuring the thickness of plaque on tooth surfaces near the gum line, using a scale from 0 to 3 to assess the oral hygiene level; ‘oral hygiene index simplified’ (OHI-S) by Greene and Vermillion^34^ for evaluating oral hygiene by scoring the amount of plaque and calculus on 6 representative teeth using a scale from 0 to 6, providing an overall measure of oral hygiene; salivary pH; salivary flow rate^36^; ‘bleeding on probing’ (BOP) for measuring gum health by recording the presence or absence of bleeding after probing the gingival sulcus, indicating the level of inflammation^37^; ‘periodontal pocket depth’ (PPD) for measuring the periodontal pocket by probing the space between the cemento-enamel-junction and the base of the gingival sulcus, reflecting the severity of periodontal disease^15^; ‘clinical attachment loss’ (CAL) is consisting of the periodontal pocket depth and also the gingival recession measured from the gingival margin to the cemento-enamel junction, indication the extent of tissue and bone destruction,^38^ ‘gingival index’ (GI) by Silness and Löe^35^ for assessing the severity of gingival inflammation by evaluating the condition of the gingiva around each tooth, using a scale from 0 to 3, while also considering the presence of bleeding focusing on the gingival tissue and the ‘community periodontal index’ (CPI)^34^ for recording and assessing the state of periodontal health in population groups, using a score from 0 to 4, with higher scores indicating more severe periodontitis.

Discrepancies between the reviewers' decisions in all phases were resolved through consultation with an additional reviewer (RBC). Extraction included the mean measurement, standard deviation and number of patients for each outcome. The mean and standard deviation were calculated using Microsoft Excel software for continuous outcomes involving patient age, number, gender, CKD stage determined by GFR, DMFT index, PI, oral hygiene index (simplified), saliva pH, stimulated and unstimulated salivary flow rate, ‘bleeding on probing’, ‘gingival index’, ‘periodontal pocket depth’, and ‘clinical attachment loss’.

As part of the data extraction process, studies that reported interquartile ranges (IQR) instead of means and SD were included by estimating these values using established statistical methods. The mean was approximated as: $Mean=\;\frac{Q1+Median+Q3}{3}$. The standard deviation was estimated using the formula: $SD=\;\frac{Q3-Q1}{1.35}$.

### Quality assessment of selected studies

To assess the risk of bias of each included study a bias analysis was performed. The quality of non-randomised studies was evaluated using the ‘Newcastle-Ottawa Scale’ (NOS), with adaptations for cross-sectional and case-control study designs, and addressed 3 quality dimensions (selection, comparability, and exposure). The following aspects were examined: the selection of study groups, the comparability between groups, and the assessment of the exposure or outcome of interest for case-control studies and cross-sectional studies^39^ (Supplementary material IV) Additionally, the ‘Risk Of Bias In Non-randomised Studies of Exposures’ (ROBINS-E) tool was used to evaluate bias in studies assessing exposure effects. The ROBINS-E tool examined 7 domains, including participant selection, exposure assessment, group comparability, and outcome measurement/reporting, with each domain assessed through signalling questions. ROBINS-E possible risk of bias judgements were ‘Low risk of bias’, ‘Some concerns’, ‘High risk of bias’, and ‘Very high risk of bias’.^40^ The primary objective of the exposure tool was used to evaluate the quality of methods to assess dental parameters of oral health in the CKD population. For assessing the quality of evidence and strength of recommendations, the GRADE (Grading of Recommendations, Assessment, Development, and Evaluation) system was approached. The GRADE methodology started by formulating a clear and specific question, ensuring all key outcomes are defined.^41^ Once evidence is gathered and summarised, GRADE used structured criteria to evaluate the quality of the evidence, considering factors such as study design, risk of bias, precision, consistency, relevance, and effect size.^41^ As suggested by GRADE a summary of findings table was conducted (Table 2). The assessment was conducted independently by 2 reviewers (A.A.A. and S.C.), who analysed the full texts of the articles.

**Table 2:** Stages of chronic kidney disease (National Kidney Foundation clinical practice guidelines).^104^

Stage of CKD	Stage description	GFR mL/min/1.73 m^2^
Stage 1	Kidney damage* with normal or high GFR	90 or higher
Stage 2	Kidney damage* and mild decrease in GFR	60-89
Stage 3	Moderate decrease	30-59
Stage 4	Severe decrease in GFR	15-29
Stage 5	Kidney failure	less than 15 or dialysis

Abbreviation: GFR, glomerular filtration rate.

⁎ Kidney damage as manifested by abnormalities noted on renal pathology, blood, urine, or imaging tests.

### Statistical analysis (meta-analysis)

The meta-analysis was conducted using the RevMan software, version 6.5.2. Means and SD were utilised to calculate the weighted mean difference and its 95% CI for DMFT, PI, oral hygiene index (simplified), saliva pH, stimulated and unstimulated salivary flow rate, bleeding on probing, GI, PPD, and CAL. A p-value below 0.05 was regarded as indicative of statistical significance. A random effects model was used to accommodate the expected heterogeneity among the studies attributable to variations in study design. Accordingly, mean differences (MD), I² statistics, and *P*-values were recorded. In the meta-analysis, only studies relating to the above-mentioned oral health-related parameters with early or advanced stages were analysed.

## Results

### Study selection

The PRISMA diagram is shown in Figure 1. A total of 1584 publications were identified through a literature search conducted in June 2025 across 4 online databases: Web of Science, PubMed, Cochrane, and ClinicalTrials.gov. After removing duplicates, 1,045 publications were screened based on their titles (Cohen’s Kappa = 0.882), of which 400 were further assessed by their abstracts (Cohen’s Kappa =0.903). In the final step, 91 publications identified as relevant for the systematic review were evaluated for eligibility based on the predefined inclusion and exclusion criteria applied to the full-text articles. Of these, 67 were excluded because they were mixed studies with patients undergoing dialysis or kidney transplantation (stage 5), studies without a healthy control group or with parameters not relevant to the review (Supplementary Table I).

Finally, 24 studies met the inclusion criteria and were included in a qualitative synthesis (Cohen’s Kappa = 0.924). Based on this, 22 studies were included in the quantitative synthesis, as the data in 2 studies were not comparable because they lacked a depiction of the standard deviation of the mean.

### Study characteristics

The characteristics of the 18 cross-sectional and 6 case-control studies are summarised in Table 3. The average age of CKD patients across all studies was 38.23 ±19.41. The overall proportion of male subjects in the CKD group was higher (60.1%) than the number of female subjects (39.9%). The classification of CKD patients into early and advanced stages is typically based on patients’ eGFR (Table 2, Table 3). The following methods were used to estimate the eGFR: Cockcroft-Gault formula (29.17%), MDRD formula (16.67%), NKF-KDOQI guidelines (12.5%), CKD-EPI equation (16.67%), BUN test (4.16%), and Schwartz formula (20.82%). Detailed information is shown in Supplementary Tables II and III.

**Table 3:** Summary of study characteristics (*n* = 24).

Study (author/year)	Country	Study design	Sample size controls (N)	Sample size CKD patients (N)	Age c/CKD	Sex c/CKD (male/female %)	Stage of CKD	Oral parameters
Tadakamadla et al 2014^48^	India	Cross-sectional study	150	74	43.14 ± 2.31/46.27 ± 1.42	n.a.	CKD stage 1-4 eGFR between >90 to 15-29 mL/min/1.73 m^2^	DMFT, gingival index, OHI-S, ‘community periodontal index’
Garcez et al 2009^43^	Spain	Case-control study	80	80	44.5 ± 8.2/44.5 ± 8.2	47.5%m 52.5%f/47.5%m & 52.5%f	CKD stage 2 eGFR between 60 and 89 mL/min/1.73 m^2^	DMFT, DI, CI, ‘periodontal pocket depth’, ‘clinical attachment loss’
Tsai et al 2022^62^	Taiwan	Cross-sectional study	1280	318	29.60±6.03/32.43±5.17	86.5% m 13.5%f/93.1%m 6.9%f	CKD stage 2-3 eGFR 60–89 mL/min/1.73 m^2^ 82.45 ± 5.76	‘Periodontal pocket depth’, BOP, ‘clinical attachment loss’, Stage of PD
Valenzuela-Narváez et al 2021^64^	Peru	Cross-sectional study	722	437	65-80 years old	total 46.1%m 53.9%f	Stage 2-3 eGFR between 60-89 and 30-59 mL/min/1.73 m^2^	‘Community periodontal index’ TN
Silva et al 2019^52^	Brazil	Cross-sectional study	100	100	13.04±2.57/13.04±2.57	67%m 33%f/67%m 33%f	Stage 1-4 eGFR between >90 to 15-29 mL/min/1.73 m^2^	DMFT, PI, DED,
Borawski et al 2006^56^	Poland	Observational study	30	38	47±10/51 ±15	62%m 38%f/47% m 53%f	Advanced eGFR n.a.	Gingival index, PBI, PI, ‘clinical attachment loss’, ‘community periodontal index’, TN
Palathingal et al 2022^53^	India	Cross-sectional study	30	30	37.63±10.26/59.27±10.90	22.2% m 77.8% f/27%m 73%f	Stage 2-4 eGFR<90 mL/min/1.73 m^2^ 64.77±18.94	PI, gingival index, ‘periodontal pocket depth’, ‘clinical attachment loss’
Andaloro et al 2018^44^	Italy	Cross-sectional study	61	65	9.34 ±2.43/9.92 ±2.75	52.5% m 47.5% f/ 53.8%m 46.2%f	Stage 3-5 eGFR<60 mL/min/1.73 m^2^	DMFT, DI, CI, OHI-S, Mgingival index, SFRS, SFRU
Kassim et al 2019^49^	Malaysia	Cross-sectional study	29	29	29.69 ±11.46/62.93±11.97	41.4%m 58.6%/ 75.9%m 24.1%f	Stage 3-5 mixed, eGFR between 15 and 59 mL/min/1.73 m^2^	DMFT
Gupta et al 2018^54^	India	Cross-sectional study	30	30	35.27–8.60/44.13 ± 13.53	63.3%m 36.6%f/63.3%m 36.6%f	Advanced eGFR n.a.	OHI-S, PI, gingival index, ‘periodontal pocket depth’, ‘clinical attachment loss’
Marinho et al 2007^12^	Portugal	Case-control study	64	50	60 ± 11/64 ± 11	46.9% m 53.1%f/46% m 54%f	Stage 3-5 eGFR < 60 mL/min/1.73 m^2^	DMFT, CI, DI, ‘periodontal pocket depth’
Davidovich et al 2009^58^	Israel	Cross-sectional study	32	25	9.2 ± 2.7/12.6 ± 5	71.9%m 28.1%f/56%m 44%f	Advanced eGFR 40.44+-3.72 mL/min/1.73 m^2^	CI, salivary pH
Sezer et al 2023^50^	Turkey	Cross-sectional study	52	62 (27/25)	9.8±2.6/9.2±3.0/11±2.5	57.7%m 42.3% f, stage 1-3 59.3%m 40.7%f, stage 4-5 45.7%m 54.3% f	Stage 1-3 mixed (n=27) eGFR between >90 to 30-59 mL/min/1.73 m^2^ stage 4-5 mixed (*n* = 35) eGFR between 15-29 to <15 mL/min/1.73 m^2^	DMFT, DI, CI, OHI-S
Brito et al 2012^61^	Brazil	Cross-sectional study	118	67/51	50±7/54±11	34.3%m 65.7% f/57% m 43% f	Advanced eGFR n.a.	BOP, ‘clinical attachment loss’, PI, PD
Davidovich et al 2005^55^	Israel	Cross-sectional study	38	22	12.±1.04/10.0±0.57	39.5%m 60.5%f/81.8%m 18.2% f	Advanced stage 4-5 eGFR< 30 mL/min/1.73 m^2^ 16.69 ± 16.26	PI, gingival index, BOP, ‘periodontal pocket depth’, ‘clinical attachment loss’,
Lamba et al 2023^45^	India	Cross-sectional study	105	70	36.08±10.71/31±10.1/41.5±9.7	63.8 % m 36.2% f/63.8% m 36.2% f	Stage 1-2 as early group eGFR >60 mL/min/1.73 m^2^ 85.96 ± 21.82 stage 3-5 as advanced eGFR<60 mL/min/1.73 m^2^ 20.50 ± 11.77	DMFT, salivary pH
Marinoski et al 2019^57^	Serbia	Cross-sectional study	25	50	54.20 ± 12.67/59.06 ± 14.30	64%m 36%f/62% m 38%f	Advanced eGFR n.a.	SFRU, saliva pH
Oyetola et al 2015^59^	Nigeria	Case-control study	90	90	n.a.	n.a.	Advanced eGFR n.a.	SFRU, SFRS
Pham et al 2018^51^	Vietnam	Cross-sectional study	109	111	48.2 ± 18/51.3 ± 12	43.1% m 56.9%f/45.9%m 54.1%f	Stage 3-5 mixed eGFR <60 mL/min/1.73m2	DMFT, SFRU, SFRS, salivary pH
Tomas et al 2008^21^	Portugal	Case-control study	64	50	60 ± 11/64 ± 11	46.9%m 53.1% f/46% m 54% f	Stage 3-4 eGFR between 60-15 mL/min/1.73m2	SFR, salivary pH
Belazelkovska et al 2014^22^	Macedonia	Cross-sectional study	20	30	n.a.	n.a.	Early stage Creatinine below 120 µmol/L	SFRU, SFRS, salivary pH
Thorman et al 2010^60^	Sweden	Cross-sectional study	40	40	n.a.	n.a.	Stage 1-3 eGFR between >90 to 30-59 mL/min/1.73 m^2^	SFRS, SFRU
Dokumacıgil et al 2025^47^	Turkey	Case-control study	40	14	11.05 ± 2.00/n.a.	57.5%m 42.5%f/n.a.	Stage 1-3 eGFR between >90 to 30-59 mL/min/1.73 m^2^	DMFT, OHI-S, SFRU, SFRS, salivary pH
Beyer et al 2025^46^	Germany	Cross-sectional study	81	31	9.67 ± 4.44/11.33 ± 5.19	n.a.	Stage 1-3 eGFR between >90 to 30-59 mL/min/1.73 m^2^	DMFT, OHI-S

Abbreviations: BOP, bleeding on probing; CAL, clinical attachment loss; CI, community periodontal index; DMFT, decayed, missing, filled teeth index; GI, gingival Index; n.a., not available; OHI-S, oral hygiene index simplified; PI, plaque index; PPD, periodontal pocket depth; SSFR, stimulated saliva flow rate; USFR, unstimulated saliva flow rate.

Evaluations were performed on the following dental parameters in both groups: dental condition (DMFT), oral hygiene (PI, OHI-S), salivary metrics (pH, stimulated and unstimulated flow rate), and periodontal status (‘bleeding on probing’, ‘periodontal pocket depth’, CAL, GI, CPI). The information of the evaluated parameters is given in Table 3.

### Risk of bias in the included studies

The methodological quality of the included studies was appraised by using the ‘Newcastle‐Ottawa Scale’ (Table 4), the ‘Risk Of Bias In Non-randomised Studies of Exposures’ (ROBINS-E) tool (Figure 1), and for the quality of evidence, the GRADE criteria (Table 5). For the assessment of NOS, the included studies received at least 4 stars, with one study achieving the maximum score of 10. On average, the studies received 6.7 stars, corresponding to a moderate bias risk. In the assessment, according to the ROBINS-E tool, 3 studies had a low risk of bias, 20 studies had some concerns, and one study had a high risk of bias (score <4).

**Table 4:** Quality assessment according to NOS (a) of included cross-sectional studies (*n* = 18) and (b) of included case-control studies (*n* = 4). Additional information is shown in supplementary material (Supplementary material IV).

(a). Methodological quality assessment of the studies by Newcastle-Ottawa Quality Assessment scale (NOS)								
Cross-sectional study	Selection				Comparability	Exposure		
References	1	2	3	4	5	6	7	Total quality score
Tadakamadla et al 2014^48^	*	*		*	*	**	*	7
Tsai et. al 2022^62^		*		**	*	**	*	7
Valenzuela-Narváez et al 2021^64^		*		*	*		*	4
Silva et al 2019^52^	*	*			*	**	*	6
Borawski et al 2007^56^	*	*		*	*	**	*	6
Palathingal et al 2022^53^	*	*		**	*		*	6
Andaloro et al 2018^44^		*		*	*		*	4
Kassim et al 2019^49^				**	*	**	*	6
Gupta et al 2018^54^	*	*		*	*	**	*	7
Davidovich et al 2009 (Esti Davidovich, Davidovits, Peretz, Shapira, & Aframian, 2009b)	*	*		**	*	**	*	9
Sezer et al 2023^50^	*	*		**	**	**	*	10
Brito et al 2012^61^	*	*		**	*	*	*	8
Davidovich et al 2005^55^	*			**	**	**	*	8
Lamba et al 2023^45^	*	*		**	**	**	*	9
Marinoski et al 2019^57^	*	*		**	**	*	*	8
Pham et al 2019^51^	*	*		**	**	**	*	9
Belazelkovska et al 2014^22^	*	*		**	*	*	*	7
Thorman et al 2010^60^	*	*		*	*		*	5

(b). Methodological quality assessment of the studies by Newcastle-Ottawa Quality Assessment scale (NOS)								
Case-control study	Selection				Comparability	Exposure		
References	1	2	3	4	5	6	7 8	Total quality score
Garcez et al 2009^43^			*	*	**	*	*	6
Marinho et al 2007^12^				*	**	*	*	5
Oyetola et al 2015^59^	*	*			**	*	*	6
Tomas et al 2008^21^	*		*	*	*	*	*	6
Beyer et al 2025^46^	*	*		*	*	*	*	6
Dokumacıgil et al 2025^47^	*	*		*		*	*	5

(a) Representative of the sample; 2: Size sample; 3: Non-respondents; 4: Ascertainment of exposure; 5: Based on design and analysis; 6: Assessment of outcome; 7: Statistical test; (b) 1: Adequate case definition; 2: Representativeness of the cases; 3: Selection of Controls; 4: Definition of Controls; 5: Based on design and analysis; 6: Assessment of exposure; 7: Assortment for cases and controls; 8: Non-response rate.

**Table 5:** GRADE summary of findings table for outcomes from CKD and healthy individuals.

						Inconsistency‡					
Outcome/Comparison	No. of studies	No. of Individuals*	Mean difference (95% CI)	Expected absolute effect	Risk of bias†	I°2 statistics	*P*-value	Indirectness§	Imprecision¶	Publication bias║	Quality of evidence (GRADE)
Unstimulated salivary flow rate	8	379/423	−0.16 ml/min (−0.22, −0.10)	Significant reduction in salivary flow rate	Some concerns	95%	<0.00001	Not serious	Not serious	Not applicable	Moderate, ⊕⊕⊕◯
Stimulated salivary flow rate	4	239/261	−0.60 ml/min (−0,91, −0.28)	Markedly reduced flow rate	Some concerns	98%	0.002	Not serious	Not serious	Not applicable	Moderate, ⊕⊕⊕◯
Plaque Index (PI)	3	82/98	0.20 (0.01, 0.40)	Slightly increased plaque index	Some concerns	96%	0.04	Not serious	Not serious	Not applicable	Moderate, ⊕⊕⊕◯
Periodontal pocket depth (PPD)	5	480/1140	0.13mm (−0.27, 0.53)	No significant change in PPD	Some concerns	98%	0.52	Serious	Serious	Not applicable	Low, ⊕⊕◯◯
Clinical attachment level (CAL)	4	450/1110	0.29mm (−0.05, 0.63)	Increased CAL	Serious	96%	0.10	Serious	Serious	Not applicable	Low⊕⊕◯◯
Bleeding on probing (BOP)	3	391/1067	7.83% (2.00, 13.65)	Marked increase in BOP	Some concerns	99%	0.008	Not serious	Not serious	Not applicable	Moderate, ⊕⊕⊕◯
Salivary pH	6	176/243	0.45 (0.21, 0.70)	Increased salivary pH	Some concerns	95%	0.0003	Not serious	Not serious	Not applicable	Moderate, ⊕⊕⊕◯
Oral hygiene Index (OHIS)	6	222/351	1.47 (0.89, 2.05)	Increased OHIS	Some concerns	95%	<0.00001	Not serious	Not serious	Not applicable	Moderate, ⊕⊕⊕◯
Gingiva Index (GI)	5	217/291	0.50 (−0.00, 1.00)	Minor differences in the gingival index	Some concerns	99%	0.05	Not serious	Serious	Not applicable	Low, ⊕⊕◯◯
DMFT Index	7	302/491	−0.72 (−2.10, 0.66)	No significant change in DMFT	Some concerns	95%	0.30	Not serious	Serious	Not applicable	Low, ⊕⊕◯◯

Abbreviations: CI, confidence interval; CKD, chronic kidney disease; GRADE, grading of recommendations assessment development and evaluation.

⁎ Number of CKD patients and control.

† Risk of bias: Considered as serious if overall half of the studies were of serious risk of overall bias.

‡ Inconsistency: Considered as serous when I^2^ statistics >70% an *P*-value of x^2^ test >.05.

§ Indirectness: Considered as serious when the applicability of findings was restricted in terms of population, exposure, comparator, or outcomes.

¶ Imprecision: Considered as serious when the total number of events was below 300 for dichotomous outcomes or 400 for continuous outcomes.

║ Publication bias: Considered as serious if *P*-value of Begg’s funnel plot <.05. Not applicable (NA) if the funnel plot could not be constricted given the limited number of studies. Publication bias was difficult to detect, and thus no downgrading was performed.

The quality of evidence for the pooled analysis of CAL, PDD, GI, and DMFT Index was categorised as low according to the GRADE approach, whereas the pooled analysis of the other parameters was classified as moderate. Case-control and cross-sectional studies were initially rated as low-quality evidence but were upgraded to moderate in some cases due to substantial effect sizes with no plausible confounders.

### Caries experience

The DMFT index was used as it is the most widely used method in epidemiological dentistry to determine caries experience.^42^ In all 7 studies^12^^,^^43^, ^44^, ^45^, ^46^, ^47^, ^48^ that were included in the meta-analysis, a total of 302 CKD patients and 491 healthy control patients were included.

Four further studies^49^, ^50^, ^51^, ^52^ examined the DMFT index and reported a lower prevalence of caries in the CKD group without reaching the level of significance. Since none of these studies reported mean values or standard deviations, they were excluded from the meta-analysis.

**Fig. 2:**
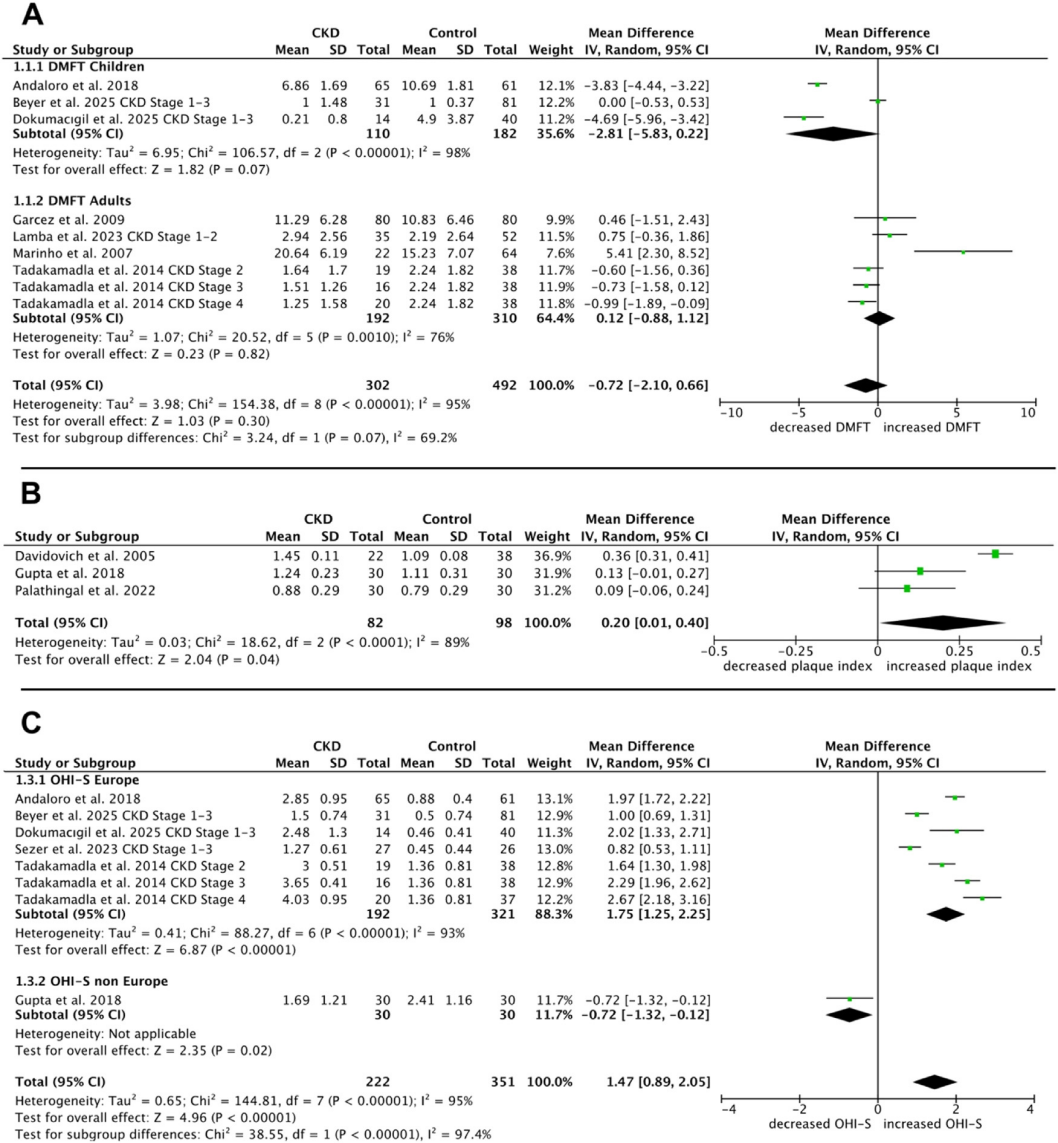
Meta-analysis of chronic kidney disease (CKD) patients versus healthy individuals (control group): A, Number of decayed, filled, or missing teeth (DMFT)—meta-analysis including a subgroup analysis of children and adults; B, Plaque Index—meta-analysis; C, Oral hygiene index simplified (OHI-S)—meta-analysis including a subgroup analysis of studies from Europe and non-Europe.

The meta-analysis for DMFT based on 7 studies showed an MD (95% CI) between CKD patients and the control group amounted to −0.72 (−2.10 to 0.66, *P* = .30) with high heterogeneity (I² = 95%) (Figure 2A). No significant difference between the groups was detectable.

### Oral hygiene

The PI and the simplified oral hygiene index were measured to assess the oral hygiene status. For assessing the PI, 3 studies^53^, ^54^, ^55^ were included, with a total of 82 patients with CKD and 98 healthy control subjects. As for the oral hygiene index, 4 studies^44^^,^^48^^,^^50^^,^^54^ were included, with a total of 222 CKD participants and 351 healthy participants. All 3 studies that focused on the PI, as well as all 6 studies examining the oral hygiene index, demonstrated that the group with CKD exhibited significantly poorer oral hygiene compared to healthy control patients.

For the PI, 2 other studies^52^^,^^56^ also reported higher plaque scores in the CKD group, but they could not be included in the meta-analysis summary due to missing means and standard deviations. The same applies to another study on the oral hygiene index,^12^ which also showed higher values in the areas of dental plaque and calculus. Based on 3 studies, the MD in plaque accumulation between patients with CKD and controls was 0.20 (95% CI: 0.01 to 0.40, *P* = .04), indicating a significantly higher PI in CKD patients (Figure 2B). Heterogeneity analysis showed a high I² value of 89%.

The pooled analysis of the simplified oral hygiene index across 6 studies revealed an MD (95% CI) of 1.47 (0.89 to 2.05, *P* < .00001) with a significantly increased index in patients with CKD compared to the control group (Figure 2C). The heterogeneity among the studies was high (I² = 95%).

### Salivary parameters

Saliva samples were obtained from a total of 176 patients with CKD and 243 control participants in 6 studies^21^^,^^22^^,^^45^^,^^47^^,^^57^^,^^58^ for saliva pH and a total of 379 CDK patients vs 423 control patients in 8 studies^21^^,^^22^^,^^44^^,^^47^^,^^51^^,^^57^^,^^59^^,^^60^ for unstimulated saliva flow rate. For the stimulated saliva flow rate, a total of 239 CKD patients vs 261 healthy controls were obtained in 4 studies.^22^^,^^44^^,^^59^^,^^60^ The CKD patients exhibited a significantly higher salivary pH compared to the control group. Additionally, both stimulated and unstimulated salivary flow rates were notably reduced in individuals with CKD. Additionally, another study^51^ reported a higher salivary pH in the CKD group. However, this study was not included in the meta-analysis due to a lack of mean and standard deviation data.

**Fig. 3:**
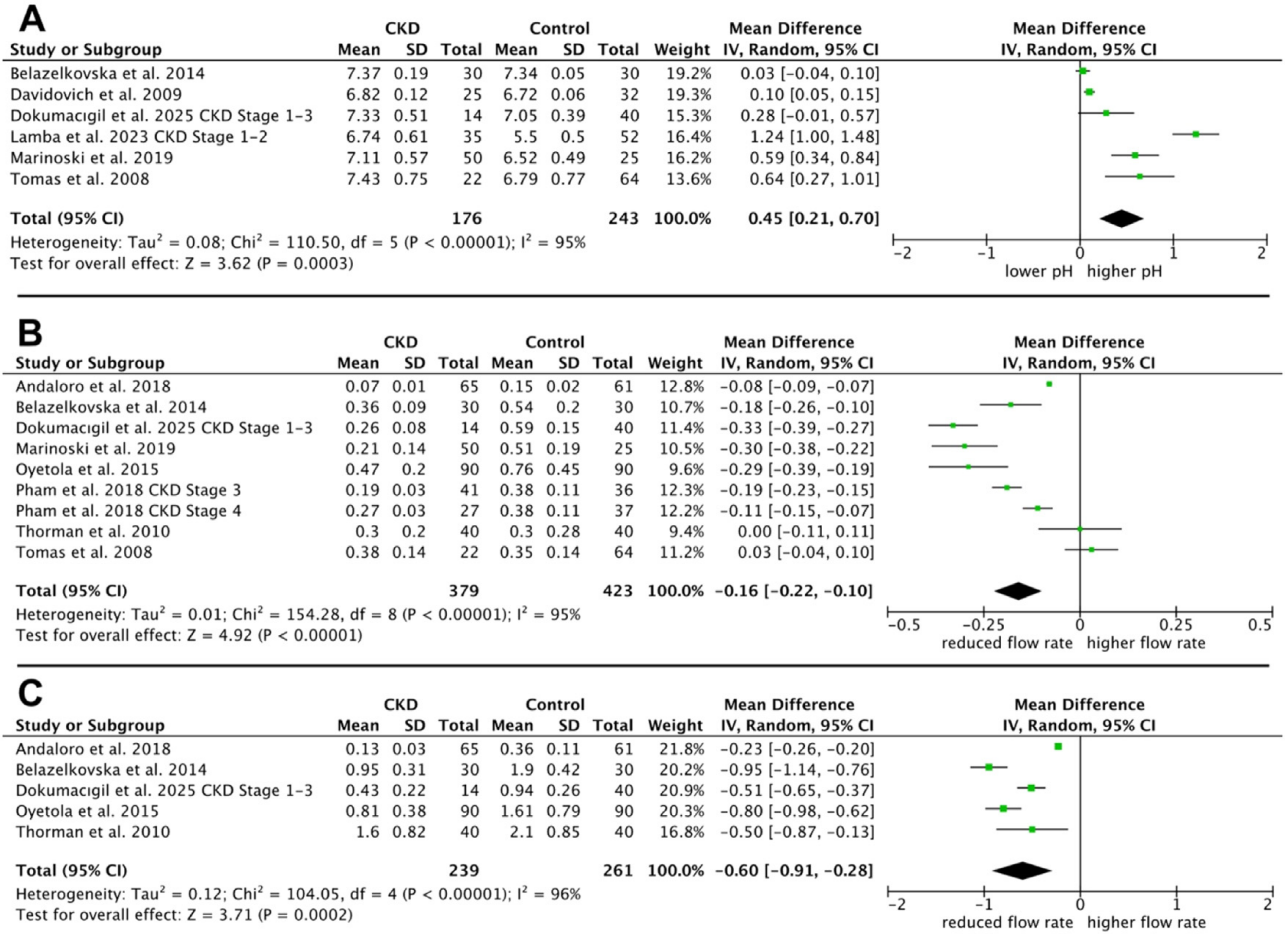
Meta-analysis of chronic kidney disease (CKD) patients versus healthy individuals (control group): A, Salivary pH; B, unstimulated salivary flow rate; C, stimulated salivary flow rate.

The meta-analysis of saliva pH showed a pooled effect size of 0.45 (95% CI: 0.21 to 0.70, *P* = .0003), indicating a significantly higher salivary pH in the CKD group compared to the control group (Figure 3A). The analysis demonstrates a high degree of heterogeneity (I² = 95%).

The meta-analysis for unstimulated salivary flow rate revealed significantly decreased flow rates in patients with CKD with an MD between CKD and control groups of −0.16 ml/min (−0.22 to −0.10, *P* < .00001) (Figure 3B). The heterogeneity among the analysed studies was high as well (I² = 95%).

The meta-analysis of stimulated salivary flow rate revealed significantly reduced flow rates in patients with early and advanced stages of CKD. The MD between the CKD and control groups was −0.60, with a 95% CI ranging from −0.91 to −0.28, and a *P*-value of .0002 (Figure 3C). The heterogeneity between the studies analysed was high, with an I² value of 96%.

### Gingival health

The parameters ‘bleeding on probing’ and ‘gingival index’ were used to assess information on the gingival health of patients with CKD. To evaluate bleeding on probing, 3 studies were included^55^^,^^61^^,^^62^, involving a total of 391 patients with CKD and 1067 healthy control subjects. As for the GI, 5 studies^43^^,^^48^^,^^53^, ^54^, ^55^ were included, with a total of 217 CKD participants and 291 healthy participants. All the above-mentioned selected studies that investigated bleeding on probing and GI demonstrated that individuals with CKD exhibited notably higher levels of gingival inflammation compared to the healthy control group. The study of Borawski et al.^56^ described an increased GI in the CKD group; however, this study was excluded from the meta-analysis due to the absence of mean and standard deviation data.

The meta-analysis for bleeding on probing, based on 3 studies,^55^^,^^61^^,^^62^ revealed significantly increased bleeding on probing values in patients with CKD with an MD (95% Cl, *P*) between CKD patients and control groups of 7.75 (2.96 to 12.54, *P* = .002) (Figure 2A) (I² = 98%).

**Fig. 4:**
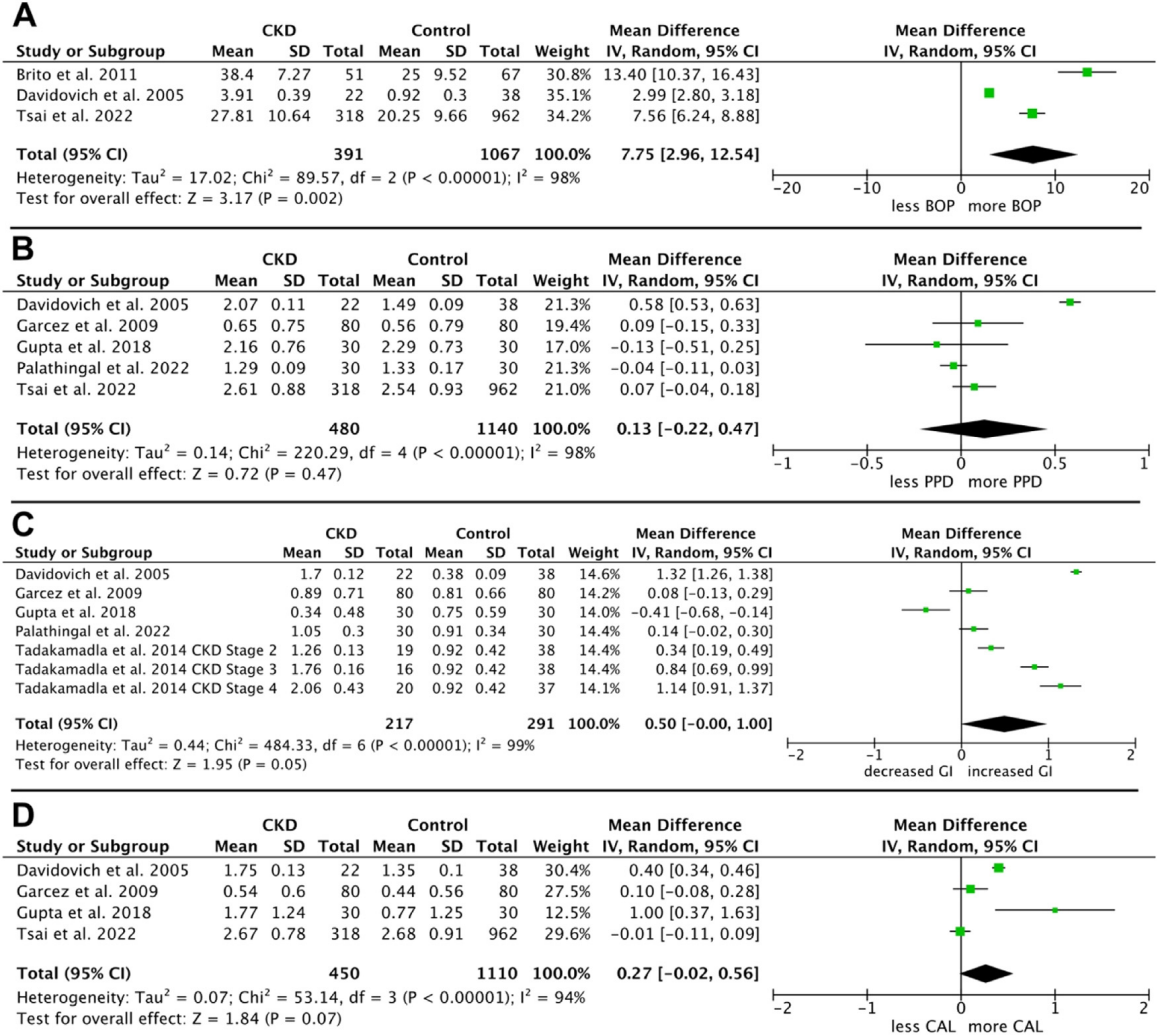
Meta-analysis of chronic kidney disease (CKD) patients versus healthy individuals (control group): A, bleeding on probing (BOP); B, gingival index; C, periodontal pocket depth (‘periodontal pocket depth’); D, clinical attachment loss (‘clinical attachment loss’).

The GI of CKD patients and healthy individuals was compared in 5 studies, and a significant increase in gingival inflammation was observed. The MD between the CKD and control groups was 0.50, with a 95% CI ranging from 0.00 to −1.0 and a *P*-value of .05 (Figure 4B). The heterogeneity among the analysed studies was high, with an I² value of 99%.

### Periodontal health

The assessment of periodontal health in patients with CKD was conducted using ‘periodontal pocket depth’, ‘clinical attachment loss’, and the ‘community periodontal index’. Five studies^43^^,^^53^, ^54^, ^55^^,^^62^ were included to evaluate PPD, comprising a total of 480 patients with CKD and 1140 healthy control subjects. As for CAL, 4 studies^52^^,^^57^^,^^58^^,^^63^ included 450 CKD participants and 1110 healthy participants. These studies focused on ‘periodontal pocket depth’, as well as all on the ‘clinical attachment loss’ and found no significant differences between the CKD at early and advanced stages vs controls. Three studies^12^^,^^56^^,^^61^ found no significant changes in ‘clinical attachment loss’ or ‘periodontal pocket depth’ in the early and advanced group, but they were excluded from the meta-analysis due to missing mean and standard deviation data. Although ‘community periodontal index’ data were quantified according to WHO standards^34^, the lack of statistical measures prevented their inclusion and comparison through the meta-analysis. Tadakamadia et al^48^ reported that 36.5% of CKD patients had a ‘community periodontal index’ score of 4, indicating severe periodontal disease, while no such cases were observed in the control group. In addition, ‘community periodontal index’ scores of 2 and 3 were common in CKD patients at early stages.

Similarly, Valenzuela-Narváez et al^64^ described a more severe periodontal disease in CKD patients, particularly those in stage 3, with significantly higher ‘community periodontal index’ scores (3 and 4). Borawski et al^56^ also observed higher ‘community periodontal index’ scores (3 and 4) in CKD patients compared to controls. None of the studies reported mean values or standard deviations for the ‘community periodontal index’; instead, the results were primarily presented as percentages or frequencies.

Based on 5 studies, the MD (95% Cl, *P*) of PPD between CKD patients vs non-renal controls resulted in 0.13 (−0.22 to 0.47, *P* = .47) with high heterogeneity (I² = 98%). There was no significant difference between the groups (Figure 4C).

**Fig. 5:**
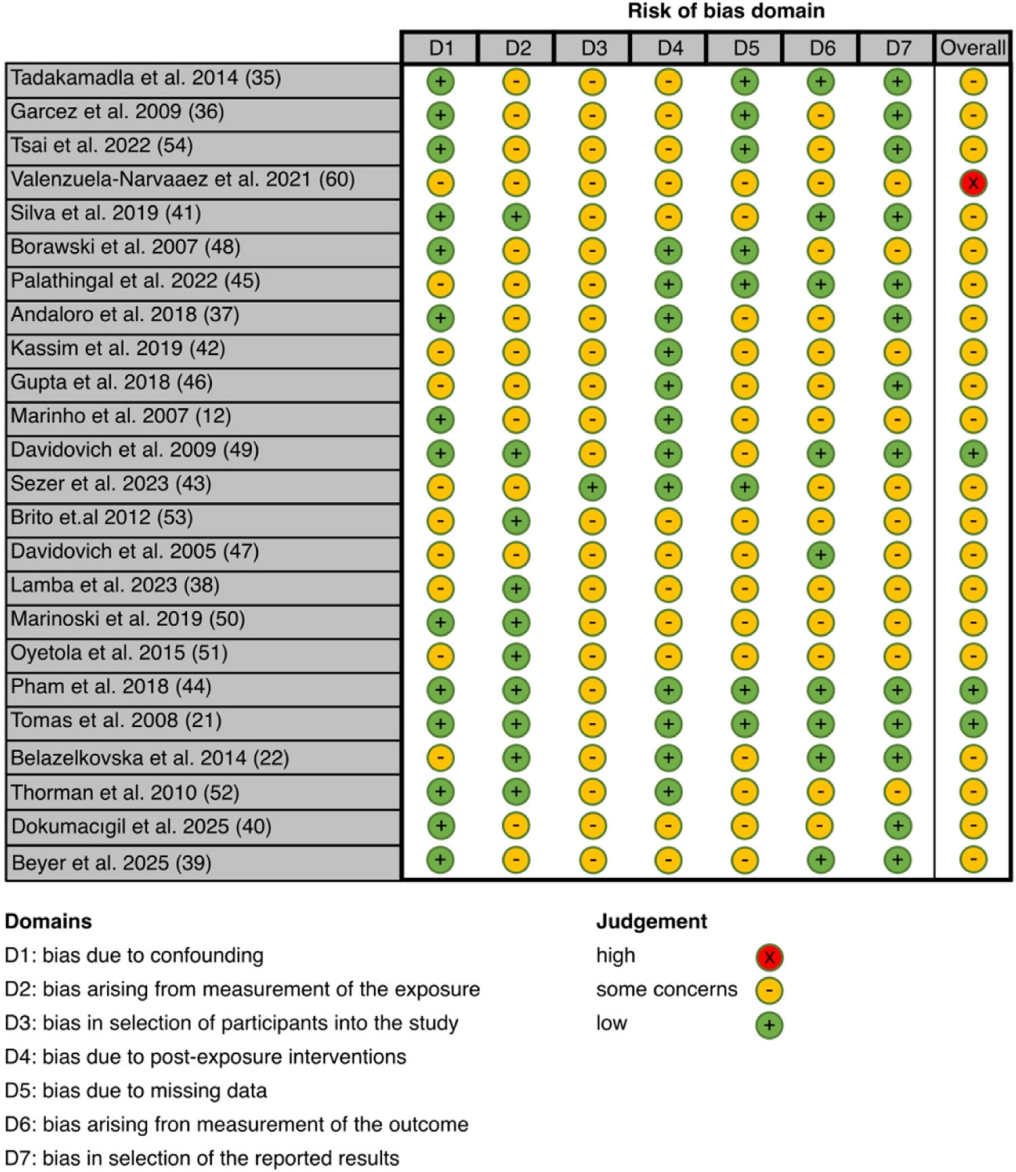
Risk of bias assessment of all included studies according to ROBINS-E tool.

The meta-analysis for CAL, based on 4 studies, revealed no significant difference with an MD (95% Cl, *P*) between CKD and the control of 0.27 (−0.02 to 0.56, *P* = .07) (Figures 4D and 5). The heterogeneity of the analysed studies was high (I² = 94%).

## Discussion

Compared to the general population, patients with CKD are at an increased risk of developing additional health complications through various mechanisms, some of which cannot easily be attributed to reduced kidney function.^65^

In the context of oral health, early recognition of a patient's status is crucial for developing an effective and personalised treatment plan aiming to prevent progressive and irreversible damage of the teeth and periodontium. End-stage CKD is already known to significantly affect oral health through altered salivary flow rate, increased plaque accumulation, and periodontal alterations.^30^ However, the influence of both early and advanced stages of CKD on periodontal remodelling remains largely unknown, including how different stages of CKD contribute to alterations in periodontal tissue structure and function. Therefore, this systematic review focuses on early as well as advanced stages of CKD, stages 1 to 4, to identify potential initial changes in oral health in these patients. The findings may provide essential insights into CKD-specific treatment strategies and serve as a valuable basis for tailoring individual treatment plans to better prevent the well-known severe periodontal and dental damages associated with an end-stage CKD.^30^^,^^66^^,^^67^

The assessment of oral health generally includes 3 components. First, the patient's oral hygiene situation. The presence of soft and hard dental plaque and tartar usually indicates a lack of regular self-care,^68^ or it may be caused by factors that make maintaining good oral hygiene at home difficult such as salivary composition and flow rate as well as patients physical inability to perform adequate oral hygiene.^69^ If plaque remains on the tooth surface for an extended period, it forms a complex biofilm that provides a habitat for pathogenic bacteria.^70^ Supported by the symbiotic effects of different bacterial strains, bacterial metabolism creates an acidic environment.^71^ This locally reduced pH leads to demineralisation of the enamel, which can clinically result in caries.^72^ The assessment of this dental damage is the second aspect of dental care. In addition to the enamel, the neighbouring soft tissues are also affected by the bacterial biofilm. The result is progressive inflammation, first as gingivitis^73^ and thereafter as localised or generalised periodontitis,^74^^,^^75^ which must be considered as the third component of oral health assessment. This leads to degenerative processes in the alveolar bone and surrounding soft tissue, which progressively deteriorates, exposing the tooth root and ultimately reducing the stability of the teeth.^76^^,^^77^ It is well known that inflammation in the periodontium can have systemic effects throughout the human body and contribute to the progression of other diseases.^78^, ^79^, ^80^, ^81^

The DMFT index quantifies the total number of carious, missing, and restored teeth or tooth surfaces. The data suggest that the oral health problems detected in the early and advanced stages of CKD have not yet led to permanent damage to the teeth. This underlines early detection as a chance to prevent loss of tooth substance to avoid long-term oral problems. Interestingly, the subgroup analysis separating children and adults revealed that DMFT is more strongly influenced in children than in adults. This may be because DMFT in children is mainly influenced by caries related to diet, hygiene, and fluoride exposure, whereas in adults it is affected by additional factors such as periodontal disease, tooth wear, and systemic health, reflecting cumulative oral damage over time.

Patients with CKD experience important changes in their saliva's flow rate and composition.^82^^,^^83^ The presented meta-analysis results on salivary pH also showed a significantly higher alkaline pH rate in CKD patients at early and advanced stages as compared to the healthy group. This may be due to urea hydrolysis, which increases ammonia levels in this population.^20^ Furthermore, the increased salivary buffering capacity in patients with CKD and the more alkaline oral pH generate an unfavourable environment for acidogenic bacteria, which may be a possible explanation for a low caries prevalence in CKD patients at early stages.^13^^,^^84^ In contrast, a low salivary pH would create an acidic environment that promotes the growth of acidogenic bacteria, leading to dental caries.^85^

The results of the simplified oral hygiene index showed an increased accumulation of debris and dental calculus in CKD patients at early and advanced stages. The general condition of CKD patients at early and advanced stages deteriorates as the disease progresses.^4^ In patients with renal pathology, a higher rate of calculus deposition is typically observed.^58^ This may be due, at least in part, to the accumulation of urea in CKD patients’ population, which increases the formation of calculus. In addition, elevated phosphate and protein levels contribute to this process.^48^^,^^86^ A recent study found that the frequency and quality of oral hygiene can reduce the impact of degenerative oral health symptoms in progressive CKD.^87^ Maintaining proper oral hygiene can also help to slow the worsening of CKD.^88^^,^^89^

CKD is associated with systemic inflammation characterised by elevated levels of inflammatory mediators such as cytokines (e.g., interleukin-6 [IL-6] and tumour necrosis factor-alpha [TNF-alpha]).^90^^,^^91^ These inflammatory markers are known to be crucial for promoting osteoclast activity,^92^^,^^93^ finally leading to alveolar bone resorption—a hallmark feature of severe periodontal disease. Furthermore, increased inflammatory oral health parameters were identified in patients at early and advanced CKD. These findings may be due to the increased accumulation of plaque and calculus in early and advanced CKD patients, as identified and explained in our analysis above. Additionally, they may be directly attributed to the systemic inflammatory effects of CKD at early and advanced stages, as patients with CKD have an increased risk of infection due to dysregulation of the immune system.^10^^,^^94^ Gingival inflammation already occurs in the early stages of CKD, indicating the need for preventive dental measures to avoid severe periodontal disease in later stages.^5^

Local plaque control reduces gingivitis in children and young adults with CKD at early and advanced stages, which also supports the need for preventive measures that systematically targeted plaque reduction.^95^ This is supported by our findings that indicate worse gingival health compared to the control group, with no significant change in periodontal health, investigated as ‘periodontal pocket depth’ and ‘clinical attachment loss’.

Many studies focusing on end-stage renal disease patients have already shown that periodontitis has developed in these patients.^9^^,^^17^^,^^96^ The progression of systemic inflammation in the progressed CKD promotes the development of periodontitis from a pre-existing gingivitis, as the initial inflammation in both conditions progresses to severe systemic inflammation, which in turn is associated with the exacerbation of the other disease.^97^ This is due to the fact that both periodontitis^98^ and CKD^99^ have multifactorial origins, with potential inflammatory mechanisms linking the 2 conditions.^6^^,^^10^^,^^94^ In addition, periodontitis has been associated with endothelial dysfunction, a mechanism also implicated in the early progression of CKD.^66^ Periodontal disease is classically characterised by progressive destruction of the soft and hard tissues of the periodontal complex.^48^ The current study clearly demonstrates that inflammatory symptoms in the oral cavity are already evident in the early stages of CKD.^94^^,^^96^^,^^100^ Our results show that CKD patients are at increased risk for gingivitis and the development of periodontitis due to altered reduced salivary flow and higher PI. The increased plaque accumulation may lead to biofilm-induced gingivitis, which results from a disruption in the symbiosis between the biofilm and the host's immune-inflammatory response, together with the development of an initial dysbiosis. This imbalance triggers an inflammatory response in the gingival tissues.^101^ For this reason, close collaboration between nephrologists and dentists is essential to reduce serious oral health problems, which are an obvious component of the late stages of CKD, through preventive dental interventions.

However, in the context of this study, some limitations must be acknowledged: First, the high heterogeneity observed in the meta-analysis may have influenced the overall findings, potentially affecting the generalizability of the results. Second, heterogeneity in meta-analyses typically occurs when the included studies differ in population, methodology or time points. The heterogeneity can be attributed to the fact that the studied patients were at varying stages of CKD, ranging from early to advanced stages, and exhibited different degrees of severity of oral disease. Differences in disease progression, systemic conditions, and treatment regimens (such as CKD-related medication) may have further contributed to this variability. Additionally, variations in study methodologies, including diagnostic criteria and assessment tools, might have influenced the reported outcomes. The existing data did not permit to detect more specific sources of heterogeneity.

The interplay between early-stage CKD and impaired oral health is complex, involving several underlying mechanisms that may affect the behaviour of periodontal cells. One of the primary factors in this interaction is the altered composition of blood and saliva in individuals with CKD. Patients with CKD are characterised by elevated blood levels of urea due to impaired renal function.^83^ This accumulation leads to increased concentrations in saliva, which contributes to changes in the oral microenvironment and potentially exacerbates periodontal conditions. Interestingly, elevated urea concentrations are also known to occur in patients with periodontitis.^102^ Urea, specifically, can be metabolised into ammonia by urease-producing bacteria in the mouth, leading to an increase in oral pH^103^^,^^104^ and promoting plaque formation^83^, which corresponds to the findings of this meta-analysis.

While our meta-analysis did not show a significant difference in DMFT or CAL in early CKD stages, the significantly higher PI and reduced salivary flow indicate a heightened vulnerability. In the context of global oral health, these findings are particularly relevant for low- and middle-income countries where CKD prevalence is rising, and dental access is sparse. We propose an interdisciplinary management model where nephrologists refer patients to dentists for screening for early oral symptoms (xerostomia, gingival bleeding) to trigger early dental intervention. Integrating such screenings into national CKD clinical guidelines could serve as a cost-effective public health strategy to improve the quality of life and systemic health of millions of patients worldwide.

## Conclusion

Taken together, the presented data highlight the importance of early monitoring of oral health in patients with early and advanced stages of CKD which is primarily characterised by gingival inflammation (increase of ‘bleeding on probing’, ‘gingival index’, ‘clinical attachment loss’) and increased accumulation of plaque and calculus (increase of ‘plaque index’ and ‘oral hygiene index’) combined with decreased unstimulated and stimulated saliva flow rate. Close cooperation between nephrologists and dentists is therefore essential to determine patient-specific interventions and recall intervals. These may include dental check-ups and professional support with oral hygiene more than twice a year. The aim is to reduce the risk of serious oral health problems that are an obvious part of the late stages of CKD.

## Author contributions

*Conceptualisation*: C.N., R.B.C.; Collecting data: C.N., R.B.C., A.A.A., S.C.; *Data analysis*: C.N., R.B.C., A.A.A., S.C.; *Statistical analysis*: C.N., R.B.C., A.A.A., S.C.; *Data interpretation*: C.N., R.B.C., A.A.A., S.C., S.K., M.R.P., R.K., U.S.S, S.W., J.J., M.W.; *Visualisation*: A.A.A., S.C., C.N., R.B.C.; *Writing—original draft preparation*: C.N., A.A.A, S.C.; *Writing—review and editing*: A.A.A., S.C., C.N., R.B.C., S.K., M.R.P., R.K., U.S.S., S.W., J.J., M.W.; All authors have read and agreed to the published version of the manuscript.

## Funding

This project was supported by the Clinician Scientist Program of the Faculty of Medicine of RWTH Aachen University to C.N. This study was funded by grants from the Interdisciplinary Center for Clinical Research within the Faculty of Medicine at the RWTH Aachen University and the ‘Deutsche Forschungsgemeinschaft’ (DFG, German Research Foundation) Project-ID 504777725, Project-ID 559483338, SFB1739 (Project-ID:546544928) and by the Transregional Collaborative Research Centre (SFB TRR219; Project-ID 322900939; subproject C-04, S-03), INST 948/4S-1, CRU 5011 (Project-ID 445703531) and Cost-Action CA 21165, ERA-PerMed (ERA-PERMED2022-202-KidneySign).

## Conflict of interest

The authors declare that they have no known competing financial interests or personal relationships that could have appeared to influence the work reported in this paper.

## Appendix Supplementary materials

Image, application 1

## Footnotes

Supplementary material associated with this article can be found, in the online version, at doi:10.1016/j.identj.2026.109610.
