# Supplementary material for: Oral Health in Early and Advanced Stages (1-4) of Chronic Kidney Disease: A Systematic Review and Meta-Analysis
Source: Int Dent J. 2026 May 18;76(4):109610. doi: 10.1016/j.identj.2026.109610 (PMC13202571; doi:10.1016/j.identj.2026.109610)
Supplement: Supplementary file 1 [file mmc1.docx]

**Oral health in early and advanced stages of chronic kidney disease:**

**A systematic review and meta-analysis**

**Supplementary table I:** Summary of excluded studies after full-text-screening (n=67)

|  | **Reference** | **Exclude reason** |
| --- | --- | --- |
| 1 | Ajithkrishnan, C. G., & Parkar, S. M. (2012). Periodontal status in patients undergoing hemodialysis. *Indian Journal of Nephrology*, *22*(4), 246–250. https://doi.org/10.4103/0971-4065.101242 | Only dialysis patients |
| 2 | Al Nowaiser, A., Roberts, G. J., Trompeter, R. S., Wilson, M., & Lucas, V. S. (2003). Oral health in children with chronic renal failure. *Pediatric Nephrology*, *18*(1), 39–45. https://doi.org/10.1007/s00467-002-0999-7 | Dialysis and non-dialysis patients mixed |
| 3 | Almeida, P. A., Fidalgo, T. K. S., Freitas-Fernandes, L. B., Almeida, F. C. L., Souza, I. P. R., & Valente, A. P. (2017). Salivary metabolic profile of children and adolescents after hemodialysis. *Metabolomics*, *13*(11), 1–10. https://doi.org/10.1007/s11306-017-1283-y | Only dialysis patients |
| 4 | Anuradha, B. R., Katta, S., Kode, V. S., Praveena, C., Sathe, N., Sandeep, N., & Penumarty, S. (2015). Oral and salivary changes in patients with chronic kidney disease: A clinical and biochemical study. *Journal of Indian Society of Periodontology*, *19*(3), 297–301. https://doi.org/10.4103/0972-124X.154178 | Dialysis and non-dialysis patients mixed |
| 5 | Ausavarungnirun, R., Wisetsin, S., Rongkiettechakorn, N., Chaichalermsak, S., Udompol, U., & Rattanasompattikul, M. (2016). Association of dental and periodontal disease with chronic kidney disease in patients of a single, tertiary care centre in Thailand. *BMJ Open*, *6*(7), 1–8. https://doi.org/10.1136/BMJOPEN-2016-011836 | No healthy control group |
| 6 | Bayraktar, G., Kazancioglu, R., Bozfakioglu, S., Yildiz, A., & Ark, E. (2004). Evaluation of salivary parameters and dental status in adult hemodialysis patients. *Clinical Nephrology*, *62*(5), 380–383. https://doi.org/10.5414/cnp62380 | Only dialysis patients |
| 7 | Bayraktar, G., Kurtulus, I., Duraduryan, A., Cintan, S., Kazancioglu, R., Yildiz, A., Bural, C., Bozfakioglu, S., Besler, M., Trablus, S., & Issever, H. (2007). Dental and periodontal findings in hemodialysis patients. *Oral Diseases*, *13*(4), 393–397. https://doi.org/10.1111/j.1601-0825.2006.01297.x | Only dialysis patients |
| 8 | Bayraktar, G., Kurtulus, I., Kazancioglu, R., Bayramgurler, I., Cintan, S., Bural, C., Bozfakioglu, S., Besler, M., Trablus, S., Issever, H., & Yildiz, A. (2008). Evaluation of periodontal parameters in patients undergoing peritoneal dialysis or hemodialysis. *Oral Diseases*, *14*(2), 185–189. https://doi.org/10.1111/j.1601-0825.2007.01372.x | Only dialysis patients |
| 9 | Bots, C. P., Poorterman, J. H. G., Brand, H. S., Kalsbeek, H., Van Amerongen, B. M., Veerman, E. C. I., & Nieuw Amerongen, A. V. (2006). The oral health status of dentate patients with chronic renal failure undergoing dialysis therapy. *Oral Diseases*, *12*(2), 176–180. https://doi.org/10.1111/j.1601-0825.2005.01183.x | Only dialysis patients |
| 10 | Cengiz, M., Sümer, P., Cengiz, S., & Yavuz, U. (2009). The effect of the duration of the dialysis in hemodialysis patients on dental and periodontal findings. *Oral Diseases*, *15*(5), 336–341. https://doi.org/10.1111/j.1601-0825.2009.01530.x | Only dialysis patients |
| 11 | Chamani, G., Zarei, M. R., Radvar, M., Rashidfarrokhi, F., & Razazpour, F. (2009). Oral health status of dialysis patients based on their renal dialysis history in Kerman, Iran. *Oral Health & Preventive Dentistry*, *7*(3), 269–275. | Only dialysis patients |
| 12 | Chung, C. J., Wu, C. H., Hu, W. L., Shih, C. H., Liao, Y. N., & Hung, Y. C. (2023). Tongue diagnosis index of chronic kidney disease. *Biomedical Journal*, *46*(1), 170–178. https://doi.org/10.1016/j.bj.2022.02.001 | Parameters not relevant to the review |
| 13 | Dannewitz, B., Sommerer, C., Stölzel, P., Baid-Agrawal, S., Nadal, J., Bärthlein, B., Wanner, C., Eckardt, K. U., Zeier, M., Schlagenhauf, U., Krane, V., & Jockel-Schneider, Y. (2020). Status of periodontal health in German patients suffering from chronic kidney disease—Data from the GCKD study. *Journal of Clinical Periodontology*, *47*(1), 19–29. https://doi.org/10.1111/jcpe.13208 | No healthy control group |
| 14 | Dembowska, E., Jaroń, A., Rasławska-Socha, J., Gabrysz-Trybek, E., Bladowska, J., Gacek, S., & Trybek, G. (2022). The Evaluation of the Periodontal Status of Hemodialysis Patients with End-Stage Renal Disease. *Journal of Clinical Medicine*, *11*(4). https://doi.org/10.3390/jcm11040975 | Only dialysis patients |
| 15 | Dirschnabel, A. J., Martins, A. de S., Dantas, S. A. G., Ribas, M. de O., Grégio, A. M. T., Alanis, L. R. de A., Ignacio, S. A., Trevilatto, P. C., Casagrande, R. W., de Lima, A. A. S., & Machado, M. Â. N. (2011). Clinical oral findings in dialysis and kidney-transplant patients. *Quintessence International (Berlin, Germany : 1985)*, *42*(2), 127–133. | Only dialysis patients |
| 16 | Fisher, M. A., Taylor, G. W., Papapanou, P. N., Rahman, M., & Debanne, S. M. (2008). Clinical and Serologic Markers of Periodontal Infection and Chronic Kidney Disease. *Journal of Periodontology*, *79*(9), 1670–1678. https://doi.org/10.1902/jop.2008.070569 | No healthy control group |
| 17 | Fisher, M. A., Taylor, G. W., Shelton, B. J., Jamerson, K. A., Rahman, M., Ojo, A. O., & Sehgal, A. R. (2008). Periodontal Disease and Other Nontraditional Risk Factors for CKD. *American Journal of Kidney Diseases*, *51*(1), 45–52. https://doi.org/10.1053/j.ajkd.2007.09.018 | No healthy control group |
| 18 | Fregoneze, A. P., De Oliveira Lira Ortega, A., Brancher, J. A., Vargas, E. T., De Paula Meneses, R., & Bönecker, M. J. S. (2013). Sialometric analysis in young patients with chronic renal insufficiency. *Special Care in Dentistry*, *33*(3), 118–122. https://doi.org/10.1111/scd.12008 | Only dialysis patients |
| 19 | Fregoneze, A. P., Ortega, A. D. O. L., Brancher, J. A., Vargas, E. T., Braga, I. K., Gemelli, S., De Paula Ataide, A. F. G., Ignácio, S. A., & Bönecker, M. J. S. (2015). Clinical evaluation of dental treatment needs in chronic renal insufficiency patients. *Special Care in Dentistry*, *35*(2), 63–67. https://doi.org/10.1111/scd.12094 | Only dialysis patients |
| 20 | Freitas-Fernandes, L. B., Fidalgo, T. K. S., de Almeida, P. A., Souza, I. P. R., & Valente, A. P. (2021). Salivary metabolome of children and adolescents under peritoneal dialysis. *Clinical Oral Investigations*, *25*(4), 2345–2351. https://doi.org/10.1007/s00784-020-03557-1 | Only dialysis patients |
| 21 | Gavaldá, C., Bagán, J. V., Scully, C., Silvestre, F. J., Milián, M. A., & Jiménez, Y. (1999). Renal hemodialysis patients: Oral, salivary, dental and periodontal findings in 105 adult cases. *Oral Diseases*, *5*(4), 299–302. https://doi.org/10.1111/j.1601-0825.1999.tb00093.x | Only dialysis patients |
| 22 | Grubbs, V., Plantinga, L. C., Crews, D. C., Bibbins-Domingo, K., Saran, R., Heung, M., Heung, O., Patel, P., Burrows, N. R., Ernst, K. L., & Powe, N. R. (2011). Vulnerable populations and the association between periodontal and chronic kidney disease. *Clinical Journal of the American Society of Nephrology*, *6*(4), 711–717. https://doi.org/10.2215/CJN.08270910 | No healthy control group |
| 23 | Grubbs, V., Vittinghoff, E., Beck, J. D., Kshirsagar, A. V., Wang, W., Griswold, M. E., Powe, N. R., Correa, A., & Young, B. (2015). Association Between Periodontal Disease and Kidney Function Decline in African Americans: The Jackson Heart Study. *Journal of Periodontology*, *86*(10), 1126–1132. https://doi.org/10.1902/jop.2015.150195 | No healthy control group |
| 24 | Höfer, K.C., Hanna, W., Isabelle, G., Anna, G., Anne, A., Barbe, A.G., Weber, L.T., Noack, M.J., 2024. Gingivitis Control in Children, Adolescents and Young Adults With Chronic Kidney Disease by a Need-Related Programme: A Randomised Clinical Trial. Int. J. Dent. Hyg. 1–12. https://doi.org/10.1111/idh.12866 | Dialysis and non-dialysis patients mixed |
| 25 | Imirzalioglu, P., Onay, E. O., Agca, E., & Ogus, E. (2007). Dental erosion in chronic renal failure. *Clinical Oral Investigations*, *11*(2), 175–180. https://doi.org/10.1007/s00784-007-0100-9 | No healthy control group |
| 26 | Ioannidou, E., Hall, Y., Swede, H., & Himmelfarb, J. (2013). Periodontitis associated with chronic kidney disease among Mexican Americans. *Journal of Public Health Dentistry*, *73*(2), 112–119. https://doi.org/10.1111/j.1752-7325.2012.00350.x | Parameters not relevant to the review |
| 27 | Ioannidou, E., Swede, H., & Dongari-Bagtzoglou, A. (2011). Periodontitis predicts elevated C-reactive protein levels in chronic kidney disease. *Journal of Dental Research*, *90*(12), 1411–1415. https://doi.org/10.1177/0022034511423394 | Parameters not relevant to the review |
| 28 | Jabbarzadehkhoei, F., Bakhshandeh, S., Namdari, M., Pakkhesal, M., & Khoshnevisan, M. H. (2021). Periodontal and chronic kidney diseases: A modifiable association. *Journal of Contemporary Medical Sciences*, *7*(1), 23–27. https://doi.org/10.22317/jcms.v7i1.919 | No healthy control group |
| 29 | Joshi, N. P., Shrestha, A., Bhagat, T., Agrawal, S. K., & Chhetri, R. (2024). The Oral Health Condition of Patients on Hemodialysis at a Tertiary Healthcare Facility in Eastern Nepal. *International Journal of Dentistry*, *2024*. https://doi.org/10.1155/2024/3776702 | Only dialysis patients |
| 30 | Kim, Y. J., Moura, L. M. de, Caldas, C. P., Perozini, C., Ruivo, G. F., & Pallos, D. (2017). Evaluation of periodontal condition and risk in patients with chronic kidney disease on hemodialysis. *Einstein (Sao Paulo, Brazil)*, *15*(2), 173–177. https://doi.org/10.1590/S1679-45082017AO3867 | Only dialysis patients |
| 31 | Kitsou, V. K., Konstantinidis, A., & Siamopoulos, K. C. (2000). Chronic renal failure and periodontal disease. *Renal Failure*, *22*(3), 307–318. https://doi.org/10.1081/JDI-100100874 | Only dialysis patients |
| 32 | Kopić, V., Barbić, J., Petrović, S., Šahinović, I., Mihaljević, D., Kopić, A., & Bošnjak, A. (2019). Periodontal Disease in Different Stages of Chronic Kidney Disease. *Acta Clinica Croatica*, *58*(4), 709–715. https://doi.org/10.20471/acc.2019.58.04.18 | No healthy control group |
| 33 | Kosaka, S., Ohara, Y., Naito, S., Iimori, S., Kado, H., Hatta, T., Yanishi, M., Uchida, S., & Tanaka, M. (2020). Association among kidney function, frailty, and oral function in patients with chronic kidney disease: A cross-sectional study. *BMC Nephrology*, *21*(1), 1–8. https://doi.org/10.1186/s12882-020-02019-w | Parameters not relevant to the review |
| 34 | Krishnan, N., Vijay Kumar, S., Nair, A., Kavitha, R., Govind, M., & Remya, M. (2023). Oral health status of individuals affected with chronic kidney disease: A cross-sectional study. *Journal of Head and Neck Physicians and Surgeons*, *11*(1), 39–43. https://doi.org/10.4103/jhnps.jhnps_98_22 | No healthy control group |
| 35 | Kshirsagar, A. V., Moss, K. L., Elter, J. R., Beck, J. D., Offenbacher, S., & Falk, R. J. (2005). Periodontal disease is associated with renal insufficiency in the Atherosclerosis Risk in Communities (ARIC) study. *American Journal of Kidney Diseases*, *45*(4), 650–657. https://doi.org/10.1053/j.ajkd.2004.12.009 | Parameters not relevant to the review |
| 36 | Ma, C. I., Ma, R., & Pozos-guillén, F. J. G. A. D. J. (2007). *in a Fluoridated Area*. *31*(4), 31–34. | Parameters not relevant to the review |
| 37 | Maciejczyk, M., Szulimowska, J., Taranta-Janusz, K., Wasilewska, A., & Zalewska, A. (2020). Salivary gland dysfunction, protein glycooxidation and nitrosative stress in children with chronic kidney disease. *Journal of Clinical Medicine*, *9*(5), 1–18. https://doi.org/10.3390/jcm9051285 | Parameters not relevant to the review |
| 38 | Maćkowiak-Lewandowicz, K., Ostalska-Nowicka, D., Zachwieja, J., & Paszyńska, E. (2021). Differences between obese and non-obese children and adolescents regarding their oral status and blood markers of kidney diseases. *Journal of Clinical Medicine*, *10*(16). https://doi.org/10.3390/jcm10163723 | Parameters not relevant to the review |
| 39 | Maheshwari, S., Chahal, G. S., Grover, V., Rathi, M., Sharma, R., Sharma, R., & Jain, A. (2023). Impact of periodontal treatment on inflammatory oxidative stress in chronic kidney disease subjects: An interventional clinical trial. In *American journal of dentistry* (Vol. 36, Issue 1, pp. 15–20). | No fulltext available |
| 40 | Marakoglu, I., Kahraman Gursoy, U., Demirer, S., & Sezer, H. (2003). Periodontal status of chronic renal failure patients receiving hemodialysis. In *Yonsei Medical Journal* (Vol. 44, Issue 4, pp. 648–652). https://doi.org/10.3349/ymj.2003.44.4.648 | Only dialysis patients |
| 41 | Martins, C., Siqueira, W. L., De Oliveira, E., Guimarães Primo, L. S. D. S., & Nicolau, J. (2006). Salivary analysis of patients with chronic renal failure undergoing hemodialysis. *Special Care in Dentistry*, *26*(5), 205–208. https://doi.org/10.1111/j.1754-4505.2006.tb01439.x | Only dialysis patients |
| 42 | Martins, C., Siqueira, W. L., & Guimarães Primo, L. S. S. (2008). Oral and salivary flow characteristics of a group of Brazilian children and adolescents with chronic renal failure. *Pediatric Nephrology*, *23*(4), 619–624. https://doi.org/10.1007/s00467-007-0718-5 | Only dialysis patients |
| 43 | Martins, C., Siqueira, W. L., Oliveira, E., Nicolau, J., & Primo, L. G. (2012). Dental calculus formation in children and adolescents undergoing hemodialysis. *Pediatric Nephrology*, *27*(10), 1961–1966. https://doi.org/10.1007/s00467-012-2194-9 | Only dialysis patients |
| 44 | Menezes, C. R. S. D., Pereira, A. L. A., Ribeiro, C. C. C., Chaves, C. O., Guerra, R. N. M., Thomaz, É. B. A. F., Monteiro-Neto, V., & Alves, C. M. C. (2019). Is there association between chronic kidney disease and dental caries? A case-controlled study. *Medicina Oral Patologia Oral y Cirugia Bucal*, *24*(2), e211–e216. https://doi.org/10.4317/medoral.22737 | Only dialysis patients |
| 45 | Misaki, T., Fukunaga, A., Shimizu, Y., Ishikawa, A., & Nakano, K. (2019). Possible link between dental diseases and arteriosclerosis in patients on hemodialysis. *PLoS ONE*, *14*(12), 4–11. https://doi.org/10.1371/journal.pone.0225038 | Only dialysis patients |
| 46 | Munagala, K. K., Nanda, S., Chowdhary, Z., Pathivada, L., Vivekanandan, G., & Bodhi, S. (2022). Severity of Periodontal Disease in Chronic Kidney Disease Patients: A Hospital-Based Study. *Cureus*, *14*(6), 3–5. https://doi.org/10.7759/cureus.25646 | Only dialysis patients |
| 47 | Oyetola, E. O., Owotade, F. J., Agbelusi, G. A., Fatusi, O., Sanusi, A., & Adesina, O. M. (2015). Salivary Flow Rates of Nigerian Patients with Chronic Kidney Disease: A Case-control Study. *Journal of Contemporary Dental Practice*, *16*(4), 264–269. https://doi.org/10.5005/jp-journals-10024-1673 | Parameters not relevant to the review |
| 48 | Peneva, M., Anadoliiska, A., & Apostolova, D. (1989). [Dental caries with chronic renal insufficiency]. *Stomatologiia. Stomatology*, *71*(3), 6–10. | No fulltext available |
| 49 | Peterson, S. (1985). *Altered Plaque Ph in Crf*. *325*(5), 796–799. | No healthy control group |
| 50 | Rigothier, C., Catros, S., Bénard, A., Samot, J., Quintin, O., Combe, C., Larabi, I., Massy, Z., & Alvarez, J. C. (2023). Association between Dental Scores and Saliva Uremic Toxins. *Toxins*, *15*(11), 1–8. https://doi.org/10.3390/toxins15110666 | No healthy control group |
| 51 | Rojas Peña, S., Mora Muñoz, A., Gordillo Paniagua, G., & Jackson Herrerías, G. (1991). [DMF index in patients with end-stage kidney disease]. *Practica odontologica*, *12*(1), 9-11,13. | No fulltext available |
| 52 | Salimi, S., Ng, N., Seliger, S. L., & Parsa, A. (2014). Periodontal disease, renal dysfunction and heightened leukocytosis. *Nephron - Clinical Practice*, *128*(1–2), 107–114. https://doi.org/10.1159/000366445 | Parameters not relevant to the review |
| 53 | Scheutzel, P., & Ritter, W. (1989). [Alterations of teeth and jaws in children with chronic renal failure]. *Deutsche zahnarztliche Zeitschrift*, *44*(2), 115–118. | No fulltext available |
| 54 | Schütz, J. da S., de Azambuja, C. B., Cunha, G. R., Cavagni, J., Rösing, C. K., Haas, A. N., Thomé, F. S., & Fiorini, T. (2020). Association between severe periodontitis and chronic kidney disease severity in predialytic patients: A cross-sectional study. *Oral Diseases*, *26*(2), 447–456. https://doi.org/10.1111/odi.13236 | No healthy control group |
| 55 | Shiraishi, A., Yoshimura, Y., Nagano, F., & Shimazu, S. (2021). Association of impaired oral health status with chronic kidney disease in post-acute rehabilitation. *Gerodontology*, *38*(3), 300–307. https://doi.org/10.1111/ger.12527 | No healthy control group |
| 56 | Subramaniam, P., Gupta, M., & Mehta, A. (2012). Oral health status in children with renal disorders. *Journal of Clinical Pediatric Dentistry*, *37*(1), 89–93. https://doi.org/10.17796/jcpd.37.1.7l913347q0232v01 | No healthy control group |
| 57 | Sun, K., Shen, H., Liu, Y., Deng, H., Chen, H., & Song, Z. (2021). Assessment of Alveolar Bone and Periodontal Status in Peritoneal Dialysis Patients. *Frontiers in Physiology*, *12*(December), 1–10. https://doi.org/10.3389/fphys.2021.759056 | No healthy control group |
| 58 | Tasdemir, Z., Özsarı Tasdemir, F., Gürgan, C., Eroglu, E., Gunturk, I., & kocyigit, I. (2018). The effect of periodontal disease treatment in patients with continuous ambulatory peritoneal dialysis. *International Urology and Nephrology*, *50*(8), 1519–1528. https://doi.org/10.1007/s11255-018-1913-y | Only dialysis patients |
| 59 | Thorman, R., Neovius, M., & Hylander, B. (2009). Clinical findings in oral health during progression of chronic kidney disease to end-stage renal disease in a Swedish population. *Scandinavian Journal of Urology and Nephrology*, *43*(2), 154–159. https://doi.org/10.1080/00365590802464817 | No healthy control group |
| 60 | Tiwari, V., Saxena, V., Bhambhal, A., Tiwari, U., Singh, A., & Goud, S. (2013). The oral health status of patients with renal disease in central india: A preliminary study. *Journal of Renal Care*, *39*(4), 208–213. https://doi.org/10.1111/j.1755-6686.2013.12040.x | Only dialysis patients |
| 61 | Torres, S. A., Pereira, O., Hayacibara, M. F., Machado, C., Hayacibara, R. M., & Bretz, W. A. (2010). *Torres-2010-Periodontal-parameters-and-bana-tes*. *18*(3), 297–302. | Only dialysis patients |
| 62 | Trzcionka, A., Twardawa, H., Mocny-Pachońska, K., & Tanasiewicz, M. (2020). Oral cavity status of long-term hemodialized patients vs. their socio-economic status. *Medycyna Pracy*, *71*(3), 279–288. https://doi.org/10.13075/mp.5893.00948 | Only dialysis patients |
| 63 | Trzcionka, A., Twardawa, H., Mocny-Pachońska, K., & Tanasiewicz, M. (2021). Periodontal treatment needs of hemodialized patients. *Healthcare (Switzerland)*, *9*(2). https://doi.org/10.3390/healthcare9020139 | Only dialysis patients |
| 64 | Vesterinen, M., Ruokonen, H., Furuholm, J., Honkanen, E., & Meurman, J. H. (2011). Oral health in predialysis patients with emphasis on diabetic nephropathy. *Clinical Oral Investigations*, *15*(1), 99–104. https://doi.org/10.1007/s00784-009-0360-7 | No healthy control group |
| 65 | Wolff, A., Stark, H., Sarnat, H., Binderman, I., Eisenstein, B., & Drukker, A. (1985). The dental status of children with chronic renal failure. *The International Journal of Pediatric Nephrology*, *6*(2), 127–132 | No fulltext available |
| 66 | Yue, Q., Yin, F. T., Zhang, Q., Yuan, C., Ye, M. Y., Wang, X. L., Li, J. J., & Gan, Y. H. (2018). Carious status and supragingival plaque microbiota in hemodialysis patients. *PLoS ONE*, *13*(10), 1–15. https://doi.org/10.1371/journal.pone.0204674 | Only dialysis patients |
| 67 | Zhao, D., Zhang, S., Chen, X., Liu, W., Sun, N., Guo, Y., Dong, Y., Mo, A., & Yuan, Q. (2014). Evaluation of Periodontitis and Bone Loss in Patients Undergoing Hemodialysis. *Journal of Periodontology*, *85*(11), 1515–1520. https://doi.org/10.1902/jop.2014.140119 | Only dialysis patients |

**Supplementary table II:** Summary of calculation methods (see details in supplementary table III) used for the measurement of GFR

|  |  | **GFR Formula** | | | | | |
| --- | --- | --- | --- | --- | --- | --- | --- |
| **Paper** | **Reference:** | **1** | **2** | **3** | **4** | **5** | **6** |
| **1** | Tadakamadla et al. 2014 | x |  |  |  |  |  |
| **2** | Garcez et al. 2009 |  | x |  |  |  |  |
| **3** | Tsai et al. 2022 |  | x |  |  |  |  |
| **4** | Valenzuela-Narva´ez et al. 2021 |  |  | x |  |  |  |
| **5** | Silva et al. 2009 | x |  |  |  |  |  |
| **6** | Borawski et al. 2007 |  |  | x |  |  |  |
| **7** | Palathingal et al. 2022 |  |  |  | x |  |  |
| **8** | Andaloro et al. 2018 |  |  |  | x |  |  |
| **9** | Kassim et al. 2019 |  | x |  |  |  |  |
| **10** | Gupta et al. 2018 |  |  |  |  | x |  |
| **11** | Marinho et al. 2007 | x |  |  |  |  |  |
| **12** | Davidovich et al. 2009 |  |  |  |  |  | x |
| **14** | Sezer et al. 2022 |  |  |  |  |  | x |
| **15** | Brito et al. 2012 |  |  | x |  |  |  |
| **16** | Davidovich et al. 2005 |  |  |  |  |  | x |
| **17** | Lamba et al. 2023 |  |  |  | x |  |  |
| **18** | Marinoski et al. 2019 | x |  |  |  |  |  |
| **19** | Oyetola et al. 2015 | x |  |  |  |  |  |
| **20** | Pham et al. 2019 |  |  |  | x |  |  |
| **21** | Tomas et al. 2008 | x |  |  |  |  |  |
| **22** | Belazelkovska et al. 2014 | x |  |  |  |  |  |
| **23** | Thorman et al. 2010 |  | x |  |  |  |  |
| **24** | Dokumacagil et al. 2025 |  |  |  |  |  | x |
| **25** | Beyer et al. 2025 |  |  |  |  |  | x |
|  | Distrubution of the 24 inlcuded studies  in numbers | **7** | **4** | **3** | **4** | **1** | **5** |
|  | Distrubution of the 24 inlcuded studies  in per cent | **29,17%** | **16,67%** | **12,50%** | **16,67%** | **4,16%** | **20,83%** |

**Supplementary table III:** Calculation methods used for the measurement of GFR

| **Formula Number** | **Method used for GFR measurement** | **Reference** |
| --- | --- | --- |
| **1** | 1. Cockcroft Gault formula : Cockcroft-Gault formula is a method for estimating GFR based on the measurement of serum creatinine. This formula was developed in 1973 and takes into account not only serum creatinine but also the age, sex and body weight of the person. | Cockcroft, D. W., & Gault, M. H. (1976). Prediction of creatinine clearance from serum creatinine. *Nephron*, *16*(1), 31–41. https://doi.org/10.1159/000180580 |
| **2** | 2. DRD formula: Modification of Diet in Renal Disease. Takes into account age, gender, serum creatinine and ethnicity. | Lachter, J. (2023). Annals of Internal Medicine. Annals of Internal Medicine, 176(6), 461–470. https://doi.org/10.7326/L23-0065 |
| **3** | 3. The NKF KDOQI guidelines recommend using the MDRD formula to estimate GFR, especially in patients with chronic kidney disease, as it is considered more accurate and is standardized to a body surface area of 1.73 m² | K/DOQI clinical practice guidelines for chronic kidney disease: evaluation, classification, and stratification. (2002). *American Journal of Kidney Diseases : The Official Journal of the National Kidney Foundation*, *39*(2 Suppl 1), S1-266. |
| **4** | 4. The 2009 CKD-EPI equation is a method for estimating glomerular filtration rate (eGFR)  based on serum creatinine levels. This equation was developed to be more accurate than previous methods, especially at higher GFR values | Kramer, H. J., Jaar, B. G., Choi, M. J., Palevsky, P. M., Vassalotti, J. A., & Rocco, M. V. (2022). An Endorsement of the Removal of Race From GFR Estimation Equations: A Position Statement From the National Kidney Foundation Kidney Disease Outcomes Quality Initiative. *American Journal of Kidney Diseases*, *80*(6), 691–696. https://doi.org/10.1053/j.ajkd.2022.08.004 |
| **5** | 5. The BUN test measures the level of nitrogen in the blood, which is derived from urea, to assess kidney function | Hosten, A. O. (1990). *BUN and Creatinine.* (H. K. Walker, W. D. Hall, & J. W. Hurst (Eds.)). |
| **6** | 6. The Schwartz formula for calculating the glomerular filtration rate (GFR)/CHILDREN | Schwartz, G. J., Haycock, G. B., Edelmann, C. M. J., & Spitzer, A. (1976). A simple estimate of glomerular filtration rate in children derived from body length and plasma creatinine. *Pediatrics*, *58*(2), 259–263. |

**Supplementary material IV:** Quality assessment of included studies according to NOS

1. **NEWCASTLE - OTTAWA QUALITY ASSESSMENT SCALE**

**CASE CONTROL STUDIES**

Note: A study can be awarded a maximum of one star for each numbered item within the Selection and Exposure categories. A maximum of two stars can be given for Comparability.

**Selection**

1) Is the case definition adequate?

a) yes, with independent validation *****

b) yes, eg record linkage or based on self reports

c) no description

2) Representativeness of the cases

a) consecutive or obviously representative series of cases *****

b) potential for selection biases or not stated

3) Selection of Controls

a) community controls *****

b) hospital controls

c) no description

4) Definition of Controls

a) no history of disease (endpoint) *****

b) no description of source

**Comparability**

1) Comparability of cases and controls on the basis of the design or analysis

a) study controls for _______________ (Select the most important factor.) *****

b) study controls for any additional factor ***** (This criteria could be modified to indicate specific control for a second important factor.)

**Exposure**

1) Ascertainment of exposure

a) secure record (eg surgical records) *****

b) structured interview where blind to case/control status *****

c) interview not blinded to case/control status

d) written self-report or medical record only

e) no description

2) Same method of ascertainment for cases and controls

a) yes *****

b) no

3) Non-Response rate

a) same rate for both groups *****

b) non respondents described

c) rate different and no designation

1. **NEWCASTLE-OTTAWA QUALITY ASSESSMENT SCALE**

**ADAPTED FOR CROSS-SECTIONAL STUDIES***

**Selection:** (Maximum 5 stars)

1) Representativeness of the sample:

a) Truly representative of the average in the target population.***** (all subjects or random sampling)

b) Somewhat representative of the average in the target population. ***** (non-random sampling)

c) Selected group of users.

d) No description of the sampling strategy.

2) Sample size:

a) Justified and satisfactory.*****

b) Not justified.

3) Non-respondents:

a) Comparability between respondents and non-respondents characteristics is established, and the response rate is satisfactory.*****

b) The response rate is unsatisfactory, or the comparability between respondents and non-respondents is unsatisfactory.

c) No description of the response rate or the characteristics of the responders and the non-responders.

4) Ascertainment of the exposure (risk factor):

a) Validated measurement tool.******

b) Non-validated measurement tool, but the tool is available or described.*****

c) No description of the measurement tool.

**Comparability:** (Maximum 2 stars)

1) The subjects in different outcome groups are comparable, based on the study design or analysis. Confounding factors are controlled.

a) The study controls for the most important factor (select one).*****

b) The study control for any additional factor.*****

**Outcome:** (Maximum 3 stars)

1) Assessment of the outcome:

a) Independent blind assessment.******

b) Record linkage.******

c) Self report.*****

d) No description.

2) Statistical test:

a) The statistical test used to analyze the data is clearly described and appropriate, and the measurement of the association is presented, including confidence intervals and the probability level (p value).*****

b) The statistical test is not appropriate, not described or incomplete.

*This scale, as published in Herzog et al. *BMC Public Health.* 2013;**13**(1):154 has been adapted from the Newcastle-Ottawa Quality Assessment Scale for cohort studies to perform a quality assessment of cross-sectional studies for the present systematic review

**References**

1. WHO. *Oral Health Surveys - Basic Methods 5th Edition*. Vol 5. 5th ed. (Peterson PRB, ed.).; 2013.

2. Löe H, Silness J. Periodontal disease in pregnancy I. Prevalence and severity. *Acta Odontol Scand*. 1963;21(6):533-551. doi:10.3109/00016356309011240

3. Humphrey, Williamson RT. A review of saliva Normal composition, flow, and function. Humphrey, Williamson. 2001. Journal of Prosthetic Dentistry.pdf. *J Prosthet Dent*. 2001;85(2):162-169.

4. Lang NP, Joss A, Orsanic T, Gusberti FA, Siegrist BE. Bleeding on probing. A predictor for the progression of periodontal disease? *J Clin Periodontol*. 1986;13(6):590-596. doi:10.1111/j.1600-051X.1986.tb00852.x

5. Salvi GE, Roccuzzo A, Imber JC, Stähli A, Klinge B, Lang NP. Clinical periodontal diagnosis. *Periodontol 2000*. 2023;(February):1-19. doi:10.1111/prd.12487

6. Vandana K, Haneet R. Cementoenamel junction: An insight. *J Indian Soc Periodontol*. 2014;18(5):549-554. doi:10.4103/0972-124X.142437

7. Schmucker CM, Blümle A, Schell LK, et al. Systematic review finds that study data not published in full text articles have unclear impact on meta-analyses results in medical research. *PLoS One*. 2017;12(4):1-16. doi:10.1371/journal.pone.0176210

8. Higgins JPT, Morgan RL, Rooney AA, et al. A tool to assess risk of bias in non-randomized follow-up studies of exposure effects (ROBINS-E). *Environ Int*. 2024;186(June 2023). doi:10.1016/j.envint.2024.108602

9. Guyatt G, Oxman AD, Akl EA, et al. GRADE guidelines: 1. Introduction - GRADE evidence profiles and summary of findings tables. *J Clin Epidemiol*. 2011;64(4):383-394. doi:10.1016/j.jclinepi.2010.04.026

10. Review Manager (RevMan). Published online 2020.
